# The traditional uses, pharmacology, and phytochemistry of *Peucedanum praeruptorum* Dunn

**DOI:** 10.3389/fphar.2024.1352657

**Published:** 2024-04-03

**Authors:** Qiongxiao Wang, Qingmei Sun, Qinger Huang, Luping Qin, Bo Zhu

**Affiliations:** School of Pharmaceutical Sciences, Zhejiang Chinese Medical University, Hangzhou, China

**Keywords:** *Peucedanum praeruptorum*, pharmacology, phytochemistry, traditional uses, quality control

## Abstract

Bai Hua Qian Hu (Qianhu; *Peucedanum praeruptorum* Dunn) is a classical medicinal plant traditionally prescribed for respiratory ailments, including cough, pulmonary hypertension, and asthma. In this review, we summarize the research progress of the toxicology, pharmacokinetics, pharmacology, phytochemistry, botany, quality control, and traditional uses of *P. praeruptorum* in order to support future investigations into the scientific and therapeutic promise of this important medicinal plant. Information pertaining to *P. praeruptorum* was collected from scientific databases (ScienceDirect, Springer, SciFinder, PubMed, Baidu Scholar, Google Scholar, Web of Science), as well as toxicology papers from local conferences, M. Sc. and Ph.D. theses and dissertations, local magazines, classic texts on Chinese botanical drugs, and peer-reviewed journals. The Plant List (www.theplantlist.org) was utilized to verify the taxonomy of *P. praeruptorum*. *P. praeruptorum* was found to contain more than 119 distinct phytochemicals, including simple coumarins, pyranocoumarins, furanocoumarins, flavonoids, ketones, organic acids, and sterols, among others (e.g., praeruptorins A and B). Both crude plant extracts and purified metabolites of *P. praeruptorum* have been reported as treatments for hypertension, osteoporosis, Huntington’s disease, and cancer. In addition, extracts of *P. praeruptorum* are reported to exhibit diverse pharmacological activities, including osteogenic, anti-osteoclastogenic, antidepressant, neuroprotective, antitumor, and anti-inflammatory effects. Research into the pharmacology and phytochemistry of *P. praeruptorum* partially support both traditional uses and extraction methods. However, further research is required to elucidate the relationships between these metabolites, their molecular mechanisms, their structure-function roles, and their antagonistic and synergistic effects.

## Graphical Abstract

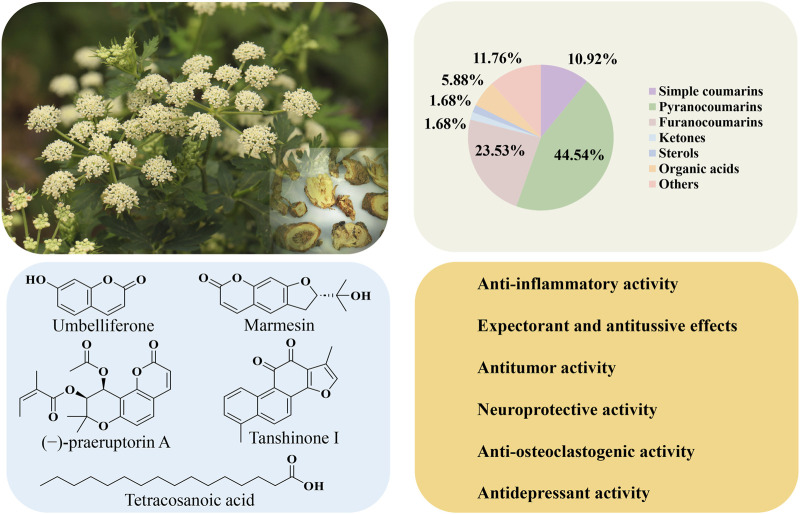

## 1 Introduction

*Peucedanum* L. (Umbelliferae) consists of 120 species of herbaceous perennial plants which are distributed widely across the globe (Editorial Committee of Flora of China, 1992). One member of the genus, *Peucedanum praeruptorum* Dunn, is cultivated in montane habitats at an altitude between 250 and 2000 m. In traditional Chinese medicine (TCM), the root tissues of *P. praeruptorum* (Qianhu) have been utilized for hundreds of years to address diverse respiratory ailments, including cough, asthma, and pulmonary hypertension (Zhou et al., 2013). The roots of *P. praeruptorum* demonstrate diverse pharmacological activities, including anti-inflammatory, neuroprotective, antitumor, anti-osteoclastogenic, antidepressant, and osteogenic effects (Song et al., 2022). In addition, *P. praeruptorum* has been found to contain an array of useful phytochemicals, including simple coumarins, pyranocoumarins, furanocoumarins, flavonoids, ketones, sterols, and organic acids, and others (Song et al., 2022).

However, to date, there has been no comprehensive and systematic evaluation of the bioactivities, pharmacology, structures, functions, and toxicities of these phytochemicals, or of *P. praeruptorum* crude extracts. Moreover, the traditional uses of *P. praeruptorum* and their pharmacological evidence have not been critically evaluated. Here, we systematically summarized the toxicology, molecular mechanisms, pharmacology, phytochemistry, botany, quality control, and traditional uses of *P. praeruptorum* to validate the medicinal use of this species. To further clarify the material basis of *P. praeruptorum*’s medicinal effect, identifying the structures of metabolites will provide a certain theoretical basis for the further development and utilization of Qianhu. The information presented here can aid the planning of clinical trials and the development of novel medicines containing *P. praeruptorum* or its active constituents.

## 2 Materials and methods

Information pertaining to *P. praeruptorum* was sourced from scientific databases (ScienceDirect, Web of Science, Springer, Google Scholar, SciFinder, PubMed, Baidu Scholar), as well as toxicology papers from local conferences, M. Sc. and Ph.D. theses and dissertations, local magazines, classic texts on Chinese botanical drugs, and peer-reviewed journals. We utilized the following, as well as related, keywords to perform the literature review: *P. praeruptorum* Dunn, secondary metabolites, toxicology, safety, ethnobotanical survey, quality control, pharmacology, medicinal uses, phytochemistry, and biological activity. The Plant List (www.theplantlist.org) was utilized to verify the taxonomy of *P. praeruptorum* and verify subspecies and cultivars. The chemical structures were drawn using ChemDraw.

## 3 Botany

**FIGURE 1:**
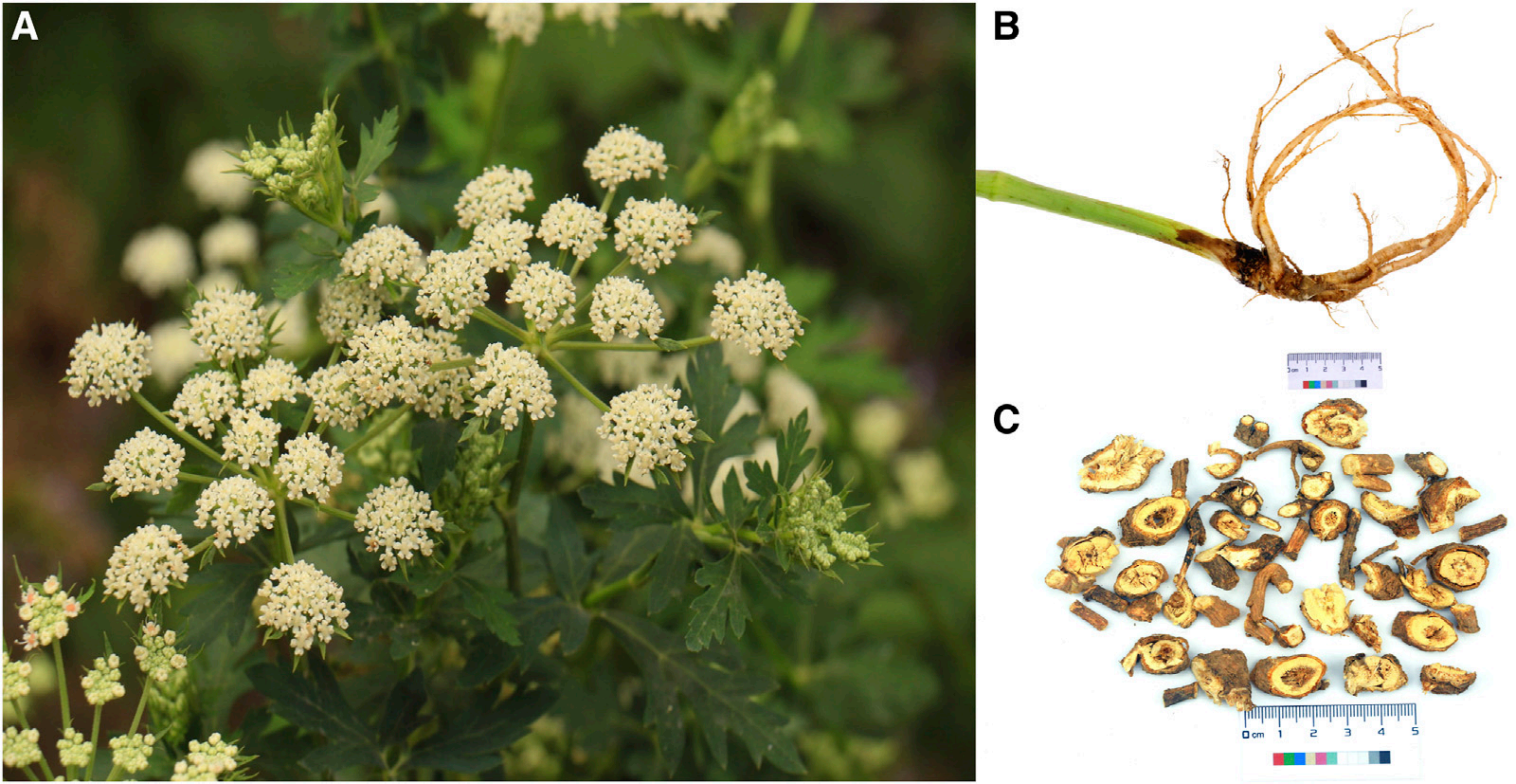
The aerial tissues **(A)**, medicinal root tissues **(B)**, and commercial presentation **(C)** of *Peucedanum praeruptorum* Dunn.

**FIGURE 2:**
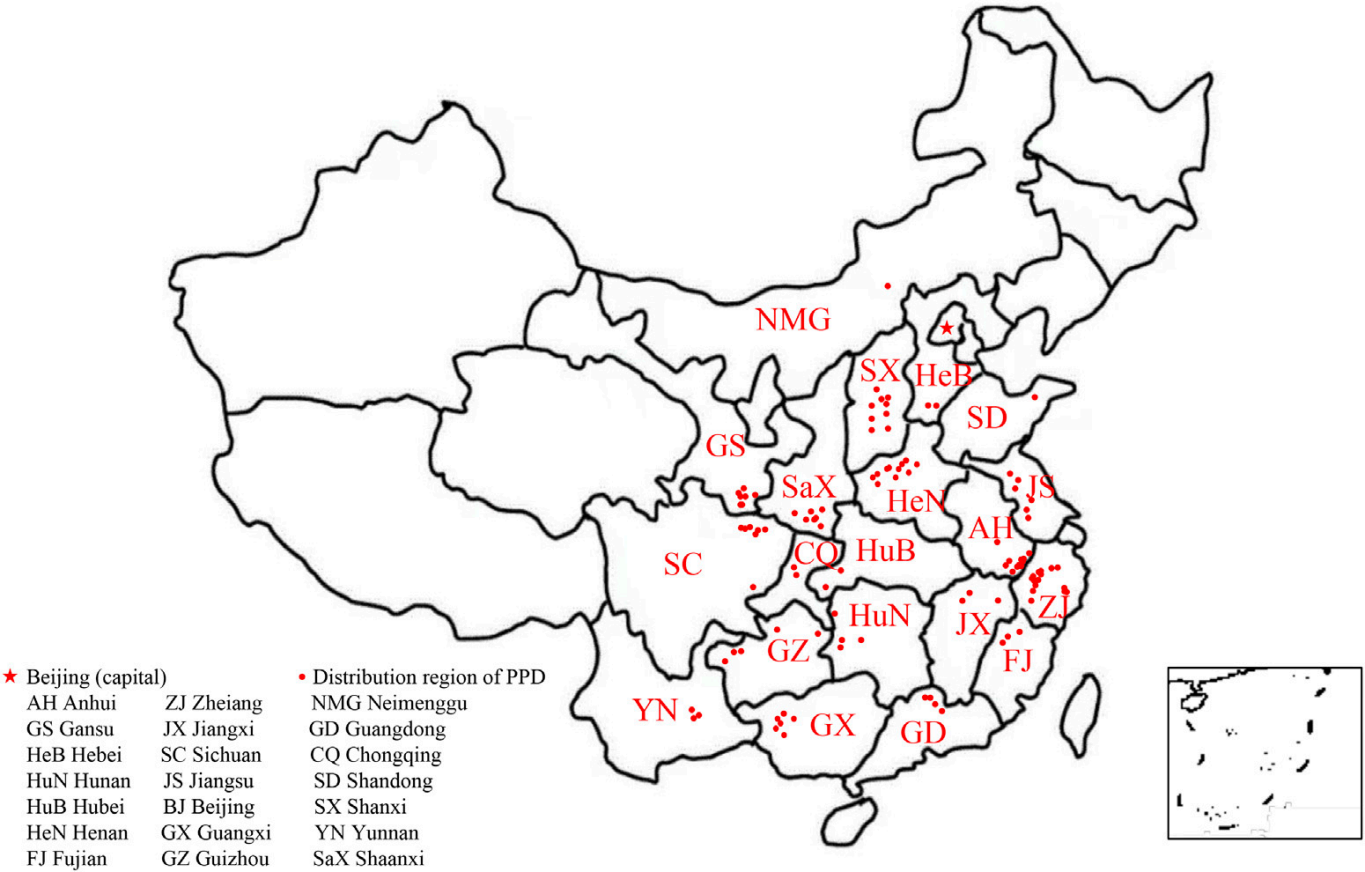
The natural distribution of *Peucedanum praeruptorum* Dunn across southern China.

*P. praeruptorum* Dunn (Figure 1) is an herbaceous perennial in the Umbelliferae family. *P. praeruptorum* Dunn is the only accepted name for the species (www.theplantlist.org), although it has two other synonyms: *P. praeruptorum* var. *grande* K.T. Fu and *P. praeruptorum* subsp. *hirsutiusculum* Ma. *P. praeruptorum* is found in the wild in south China, including in Zhejiang, Anhui, Jiangxi, Hubei, Hunan, Guizhou, Sichuan, and Yunnan provinces (Figure 2). The traditional production areas are northwest Zhejiang, southeast Anhui, and northeast Jiangxi, where the plant is called “Zhe Qianhu,” “Ning Qianhu,” and “Xin Qianhu,” respectively (Zhou et al., 2021).

According to the Flora of China (Editorial Committee of Flora of China, 1992), *P. praeruptorum* grows along forest edges, near roadsides, and in semi-open grassy areas within montane habitats at an altitude of 250–2000 m (Liu et al., 2021). *P. praeruptorum* can reach a height of 60–100 cm. The plant has a cylindrical stem, with a glabrous lower part and piliferous branches on the upper part. The medulla is solid. Leaves are wide ovate or triangular ovate. The compound umbel is terminal or lateral, and 3.5–9 cm in diameter. The fruits are oval, 4 mm in length and 3 mm in width. The back of the fruit is flat. The rhizomes are strong, brown, and 1–1.5 cm in diameter. The roots are conical and branched, with thin root ends. For medicinal use, roots are collected from winter to spring. Abluent, fresh, thin slices are dried prior to medicinal use.

## 4 Traditional uses

The List of Mingyi Bielu (《名医别录》), which dates to the Wei-Jin and South-North Dynasties (A.D. 220-450), was the first to record the roots of *P. praeruptorum* as TCM. Many ancient texts, such as the *Rihuazi Bencao* (《日华子本草》) (Five Dynasties, A.D. 908–923), the *Compendium of Materia Medica* (《本草纲目》) (Ming Dynasty, A.D. 1578), and the *Illustrated Classics of Materia Medica* (《本草图经》) (Song Dynasty, A.D. 1061) also record that *P. praeruptorum* was widely used to treat colds, headaches, coughs, asthma, and chest congestion (He et al., 2007). In the Chinese Pharmacopoeia 2000 (Chinese Pharmacopoeia Committee of People’s Repulic of China, 2000), Qianhu is defined as the roots of either *P*. *decursivum* (Miq.) Maxim (Zihuaqianhu) or *P. praeruptorum* Dunn. However, *P*. *decursivum* is not traditionally used as a source of Qianhu, and thus, was removed from the Chinese Pharmacopoeia 2005 (Chinese Pharmacopoeia Committee of People’s Repulic of China, 2005). More recently, the Chinese Pharmacopoeia 2010, 2015, and 2020 (Chinese Pharmacopoeia Committee of People’s Repulic of China, 2010; Chinese Pharmacopoeia Committee of People’s Repulic of China, 2015; Chinese Pharmacopoeia Committee of People’s Repulic of China, 2020) define Qianhu as the roots of *P. praeruptorum* exclusively, while Zihuaqianhu is defined as the roots of *P*. *decursivum*.

*P. praeruptorum* has many folk names, including *yimacai, luoguicai, shuiqianhu, shuifangfeng, shanyuansui, guanqianhu,* and *shanduhuo*. In TCM, Qianhu is used to treat chronic respiratory failure, acute bronchitis, and iridocyclitis after cataract surgery (Wang, 2016). The roots of *P. praeruptorum* have been utilized in a variety of traditional preparations, and are often used in combination with *Mentha haplocalyx* Briq., *Arctium lappa* L., and *Platycodon grandiflorum* (Jacq.) A. DC. to treat external wind-heat, body heat, headache, cough, and phlegm. In addition, *P. praeruptorum* is used in combination with *Citrus reticulata* Blanco, *Pinellia ternata* (Thunb.) Breit., and *Prunus armeniaca* L. var. *ansu* Maxim. to treat cough, chest congestion, vomiting, and nausea. The roots of *P. praeruptorum* have been used in more than 189 TCM preparations and 525 classical prescriptions (https://db.yaozh.com, accessed 7th December, 2023). Examples of TCM prescriptions containing *P. praeruptorum* are listed in Table 1. “*Bai He Qian Hu Tang*,” “*Da Qian Hu Tang*,” “*Fu Ling Qian Hu Tang*,” “*Jiu Wei Qian Hu Tang*,” and “*Xing Su San*” are Chinese classical prescriptions recorded in many ancient books. The botanical drugs “*Tong Xuan Li Fei Ke Li*” and “*Tong Xuan Li Fei Pian*” (Chinese Pharmacopoeia Committee of People’s Repulic of China, 2020), which are accredited by the National Medical Products Administration, are produced and marketed in China to treat cough. However, further research is required to clarify any potential synergisms or interactions between the bioactive phytochemicals in *P. praeruptorum* and those of other medicinal plants, as well as to elucidate their mechanisms of action. According to the Chinese Pharmacopoeia 2020 (Chinese Pharmacopoeia Committee of People’s Repulic of China, 2020), although Qianhu can disperse wind-heat, reduce cough and phlegm, and dissipate adverse Qi, comprehensive studies of its constitutive bioactive monomers should be conducted.

**TABLE 1:** Traditional Chinese medicine (TCM) prescriptions utilizing *Peucedanum praeruptorum* Dunn.

Prescription	Ingredients	Role played by qianhu in formulation	Clinical and traditional uses	References
Jin Fei Cao San	Inula japonica Thunb., Ephedra sinica Stapf, Peucedanum praeruptorum Dunn, Schizonepeta tenuisfolia Briq., Glycyrrhiza uralensis Fisch., Pinellia ternate (Thunb.) Breit., Paeonia lactiflora Pall	Leading role	Phlegmatic heat cough, wind-heat cough	Bojifang (《博济方》)
Bai He Qian Hu Tang	Lilium lancifolium Thunb., Peucedanum praeruptorum Dunn, Ephedra sinica Stapf, Pueraria lobata (Willd.) Ohwi, Ophiopogon japonicus (L.f.) KerGawl., CaSO4·2H2O	Leading role	Wind-heat cough	General Medical Collection of Royal Benevolence (《圣济总录》)
Da Qian Hu Tang	Peucedanum praeruptorum Dunn, Zingiber officinale Rosc., Pinellia ternata (Thunb.) Breit., Ephedra sinica Stapf, Scutellaria baicalensis Georgi, Paeonia lactiflora Pall., Ziziphus jujuba Mill., Citrus aurantium L	Leading role	Wind-heat cough	Waitai Miyao (《外台秘要》), Gujin Luyan (《古今录验》)
Fu Ling Qian Hu Tang	Poria cocos (Schw.) Wolf, Peucedanum praeruptorum Dunn, Chrysanthemum morifolium Ramat., Atractylodes macrocephala Koidz., Aconitum carmichaelii Debx., Asarum heterotropoides Fr. Schmidt var. mandshuricum (Maxim.) Kitag, Ephedra sinica Stapf	Leading role	Phlegmatic heat cough, wind-heat cough	General Medical Collection of Royal Benevolence (《圣济总录》)
Jia Wei Qian Hu Tang	Glycyrrhiza uralensis Fisch., Platycodon grandiflorum (Jacq.) A.DC., Morus alba L., Eriobotrya japonica (Thunb.) Lindl., Tussilago farfara L., Prunus armeniaca L.var.ansu Maxim., *Lonicera japonica* Thunb., Scutellaria baicalensis Georgi, Ophiopogon japonicus (L.f) Ker-Gawl., Anemarrhena asphodeloides Bge., Peucedanum praeruptorum Dunn	Leading role	Phlegmatic heat cough, wind-heat cough	Zhengqiaofang (《郑侨方》)
Jiu Wei Qian Hu Tang	Crataegus pinnatifida Bge. var. major N. E. Br., Schizonepeta tenuifolia Briq., Carthamus tinctorius L., Citrus aurantium L., Prunus armeniaca L.var.ansu Maxim., Platycodon grandiflorum (Jacq.) A.DC., Angelica sinensis (Oliv.) Diels, Saposhnikovia divaricata (Turcz.) Schischk., Peucedanum praeruptorum Dunn	Leading role	Phlegmatic heat cough	Zhizhen Quanshu (《治疹全书》)
Jie Geng Qian Hu Tang	Platycodon grandiflorum (Jacq.) A.DC., Perilla frutescens (L.) Britt., Prunus armeniaca L.var.ansu Maxim., Peucedanum praeruptorum Dunn, Paeonia lactiflora Pall., Morus alba L., Glycyrrhiza uralensis Fisch., Citrus reticulata Blanco, Bambusa tuldoides Munro	Leading role	Phlegmatic heat cough	Bi Hua Yi Jing (《笔花医镜》)
Pi Pa Ye Qian Hu San	Citrus aurantium L., Trionyx sinensis Wiegmann, Paeonia suffruticosa Andr., Glycyrrhiza uralensis Fisch., Paeonia lactiflora Pall., Angelica sinensis (Oliv.) Diels, Zingiber officinale Rosc., Magnolia officinalis Rehd.et Wils., Saposhnikovia divaricata (Turcz.) Schischk., Atractylodes macrocephala Koidz., Schisandra chinensis (Turcz.) Baill., Peucedanum praeruptorum Dunn, Zingiber officinale Rosc., Eriobotrya japonica (Thunb.) Lindl., Poria cocos (Schw.) Wolf, Angelica dahurica (Fisch.ex Hoffm.) Benth.et Hook.f., Pinellia ternate (Thunb.) Breit., Anemarrhena asphodeloides Bge. Pogostemon cablin (Blanco) Benth., Panax ginseng C. A. Mey., Alisma orientale (Sam.) Juzep., Platycodon grandiflorum (Jacq.) A.DC., Aucklandia lappa Decne., Areca catechu L., Akebia quinata (Thunb.) Decne., Scirpus yagara Ohwi, Terminalia chebula Retz	Leading role	Phlegmatic heat cough, wind-heat cough	Chuanjia Mibao (《传家秘宝》)
Qian Hu San	Peucedanum praeruptorum Dunn, Scutellaria baicalensis Georgi, Gardenia jasminoides Ellis, Saposhnikovia divaricata (Turcz.) Schischk., Chrysanthemum morifolium Ramat., Adenophora stricta Miq., Glycyrrhiza uralensis Fisch., Saiga tatarica Linnaeus, Ophiopogon japonicus (L.f.) KerGawl., Citrus aurantium L., CaSO4·2H2O	Leading role	Phlegmatic heat cough, wind-heat cough	Qixiao Liangfang (《奇效良方》)
Zhi Qiao Qian Hu Tang	Platycodon grandiflorum (Jacq.) A.DC., Glycyrrhiza uralensis Fisch., Peucedanum praeruptorum Dunn, Saposhnikovia divaricata (Turcz.) Schischk., Citrus aurantium L., Poria cocos (Schw.) Wolf, Perilla frutescens (L.) Britt	Leading role	Phlegmatic heat cough, wind-heat cough	Make Huoren Quanshu (《麻科活人全书》)
Xing Su San	Citrus reticulata Blanco, Prunus armeniaca L.var.ansu Maxim., Ziziphus jujuba Mill., Platycodon grandiflorum (Jacq.) A.DC., Zingiber officinale Rosc., Citrus aurantium L., Peucedanum praeruptorum Dunn, Poria cocos (Schw.) Wolf, Pinellia ternate (Thunb.) Breit., Glycyrrhiza uralensis Fisch., Perilla frutescens (L.) Britt	Leading role	Phlegmatic heat cough, wind-heat cough	Detailed analysis of epidemic warm diseases (《温病条辨》)
Jie Ji Tou Sha Tang	Forsythia suspensa (Thunb.) Vahl, Schizonepeta tenuisfolia Briq., Peucedanum praeruptorum Dunn, Glycine max (L.) Merr., Arctium lappa L., Bambusa tuldoides Munro, Cryptotympana pustulata Fabricius, Belamcanda chinensis (L.) DC., Platycodon grandiflorum (Jacq.) A.DC., Glycyrrhiza uralensis Fisch., Pueraria lobata (Willd.) Ohwi, Lasiosphaera fenzlii Reich., *Bombyx mori* Linnaeus, Spirodela polyrrhiza (L.) Schleid	Leading role	Phlegmatic heat cough, wind-heat cough	Dinshi Yian (《丁氏医案》)
Bai Du San	Mentha haplocalyx Briq., Panax ginseng C. A. Mey., Notopterygium incisum Ting ex H. T. Chang, Zingiber officinale Rosc., Angelica pubescens Maxim.f. biserrata Shan et Yuan, Glycyrrhiza uralensis Fisch., Citrus aurantium L., Poria cocos (Schw.) Wolf, Ligusticum chuanxiong Hort., Peucedanum praeruptorum Dunn, Bupleurum chinense DC., Platycodon grandiflorum (Jacq.) A.DC	Leading role	Phlegmatic heat cough, wind-heat cough	Taiping Huimin Hejiju Fang (《太平惠民和剂局方》), Direct Formula of Pediatric Medicine Syndrome (《小儿药证直诀》)
Shen Su Yin	Citrus reticulata Blanco, Aucklandia lappa Decne., Platycodon grandiflorum (Jacq.) A.DC., Poria cocos (Schw.) Wolf, Peucedanum praeruptorum Dunn, Pinellia ternate (Thunb.) Breit., Citrus aurantium L., Pueraria lobata (Willd.) Ohwi, Perilla frutescens (L.) Britt., Glycyrrhiza uralensis Fisch., Panax ginseng C. A. Mey	Leading role	Phlegmatic heat cough, wind-heat cough	Taiping Huimin Hejiju Fang (《太平惠民和剂局方》)
Cang Lin San	Notopterygium incisum Ting ex H. T. Chang, Panax ginseng C. A. Mey., Mentha haplocalyx Briq., Zingiber officinale Rosc., Oryza sativa L., Citrus aurantium L., Poria cocos (Schw.) Wolf, Angelica pubescens Maxim.f. biserrata Shan et Yuan, Ligusticum chuanxiong Hort., Bupleurum chinense DC., Peucedanum praeruptorum Dunn, Platycodon grandiflorum (Jacq.) A.DC., Glycyrrhiza uralensis Fisch	Leading role	Phlegmatic heat cough, wind-heat cough	Prescriptions for Universal Relief (《普济方》)
Qing Yan Shuang He Yin	Schizonepeta tenuisfolia Briq., Pueraria lobata (Willd.) Ohwi, *Lonicera japonica* Thunb., Platycodon grandiflorum (Jacq.) A.DC., Peucedanum praeruptorum Dunn, Glycyrrhiza uralensis Fisch., Juncus effusus L., Poria cocos (Schw.) Wolf, Scrophularia ningpoensis Hemsl., Fritillaria cirrhosa D.Don, Paeonia suffruticosa Andr., Paeonia lactiflora Pall., Angelica sinensis (Oliv.) Diels, Rehmannia glutinosa Libosch	Leading role	Phlegmatic heat cough, wind-heat cough	Houke Zizhen Ji (《喉科紫珍集》)
Jing Fang Bai Du San	Ligusticum chuanxiong Hort., Platycodon grandiflorum (Jacq.) A.DC., Saposhnikovia divaricata (Turcz.) Schischk., Glycyrrhiza uralensis Fisch., Schizonepeta tenuisfolia Briq., Poria cocos (Schw.) Wolf, Citrus aurantium L., Angelica pubescens Maxim.f. biserrata Shan et Yuan, Notopterygium incisum Ting ex H. T. Chang, Peucedanum praeruptorum Dunn, Bupleurum chinense DC.	Leading role	Plegmatic heat cough, wind-heat cough	Shesheng Zongmiao Fang (《摄生众妙方》)
Xuan Du Fa Biao Tang	Mentha haplocalyx Briq., Zingiber officinale Rosc., Oryza sativa L., Bupleurum chinense DC., Citrus aurantium L., Platycodon grandiflorum (Jacq.) A.DC., Notopterygium incisum Ting ex H. T. Chang, Ligusticum chuanxiong Hort., Panax ginseng C. A. Mey., Peucedanum praeruptorum Dunn, Glycyrrhiza uralensis Fisch., Angelica pubescens Maxim.f. biserrata Shan et Yuan, Poria cocos (Schw.) Wolf	Supporting role	Phlegmatic heat cough, wind-heat cough	Golden Mirror of Medicine (《医宗金鉴》)
Su Zi Jiang Qi Tang	Zingiber officinale Rosc., Perilla frutescens (L.) Britt., Pinellia ternate (Thunb.) Breit., Ziziphus jujuba Mill., Citrus reticulata Blanco, Magnolia officinalis Rehd.et Wils., Cinnamomum cassia Presl, Peucedanum praeruptorum Dunn, Glycyrrhiza uralensis Fisch., Angelica sinensis (Oliv.) Diels	Supporting role	Phlegmatic heat cough, wind-heat cough	Taiping Huimin Hejiju Fang (《太平惠民和剂局方》)
Qiang Huo Sheng Feng Tang	Scutellaria baicalensis Georgi., Atractylodes macrocephala Koidz., Bupleurum chinense DC., Glycyrrhiza uralensis Fisch., Citrus aurantium L., Schizonepeta tenuisfolia Briq., Notopterygium incisum Ting ex H. T. Chang, Angelica dahurica (Fisch.ex Hoffm.) Benth.et Hook.f., Mentha haplocalyx Briq., Platycodon grandiflorum (Jacq.) A.DC., Peucedanum praeruptorum Dunn, Saposhnikovia divaricate (Turcz.) Schischk., Angelica pubescens Maxim.f. biserrata Shan et Yuan, Ligusticum chuanxiong Hort	Supporting role	Phlegmatic heat cough, wind-heat cough	Yuanji Qiwei (《原机启微》)

## 5 Phytochemistry

*P. praeruptorum* is reported to contain a diverse array of phytochemicals, including simple coumarins (**1**–**13**), pyranocoumarins (**14**–**66**), furanocoumarins (**67**–**94**), ketones (**95, 96**), sterols (**97, 98**), and organic acids (**99**–**105**), and others (**106**–**119**) (Table 2). The majority of these phytochemicals were isolated from root tissues, which are the traditional medicinal material. Among these isolated metabolites, angular pyranocoumarins (e.g., praeruptorins A and B) are the most abundant bioactive metabolites in *P. praeruptorum* tissues (Song et al., 2015).

**TABLE 2:** Phytochemicals isolated from *Peucedanum praeruptorum* Dunn.

Molecular class	Phytochemical	Tissue	Identification and isolation methods^a^	Extract type	References
Simple coumarins	Umbelliferone **1**	Root	MRCC, NMR, OCC, MS, SGCC	Ethanol	Zhang et al. (2011)
	Scopoletin **2**	Root	HPLC, HREIMS, IR, UV, NMR, SGCC	Petroleum ether	Kong et al. (1994b)
	Isoscopoletin **3**	Root	MRCC, SGCC, OCC, NMR, MS	Ethanol	Zhang et al. (2011)
	Isofraxidin **4**	Root	OHPLCc18/c30, NMR, EIMS	Water	Ishii et al. (2008)
	8-carboxy-7-hydroxy coumarin **5**	Root	OHPLCc18/c30, NMR, EIMS	Water	Ishii et al. (2008)
	Skimmin **6**	Root	HPLC, NMR, EIMS	N-butanol	Okuyama et al. (1989)
	Scopolin **7**	Root	MS, UHPLC/ToFMS	Methanol	Chen et al. (2019)
	Osthenol **8**	Root	HPLC	Ethanol	Chen et al. (2021)
	Praeroside VI **9**	Root	EIMS, OHPLCc18/c30, NMR	Water	Ishii et al. (2008)
	Apiosylskimmin **10**	Root	EIMS, OHPLCc18/c30, NMR	Water	Ishii et al. (2008)
	Hymexelsin **11**	Root	EIMS, OHPLCc18/c30, NMR	Water	Ishii et al. (2008)
	Eleutheroside B1 **12**	Root	MS, NMR, OCC, SGCC	Ethanol	Zhang et al. (2009)
	(−)-peucedanol **13**	Root	EIMS, NMR, SGCC	Petroleum ether	Kong et al. (1993a)
Pyranocoumarin	Praeruptorin C **14**	Root	FC/ACC, NMR	Diethyl ether/petroleum ether	Chen et al. (1979)
	Praeruptorin E **15**	Root	MS, UHPLC/ToFMS	Methanol	Chen et al. (2019)
	Qianhucoumarin D **16**	Root	MS, UHPLC/ToFMS	Methanol	Chen et al. (2019)
	Qianhucoumarin A **17**	Root	MS, UHPLC/ToFMS	Methanol	Chen et al. (2019)
	Khellactone **18**	Root	MS, UHPLC/ToFMS	Methanol	Chen et al. (2019)
	Qianhucoumarin B **19**	Root	MS, UHPLC/ToFMS	Methanol	Chen et al. (2019)
	(9R,10R)-9-acetoxy-8,8-dimethyl-9,10-dihydro-2H,8H-benzo [1,2-b:3,4-b’] dipyran-2-one-10-yl-ester **20**	Root	HPLC	Ethanol	Chen et al. (2021)
	(±) cis-4′-acetyl-3′-crotonoykhellactone **21**	Root	MS, HPLC, NMR	Ethanol	Chen et al. (2021)
	Qianhucoumarin E **22**	Root	UHPLC/ToFMS, MS	Methanol	Chen et al. (2019)
	Hyuganin D **23**	Root	UHPLC/ToFMS, MS	Methanol	Chen et al. (2019)
	Qianhucoumarin I **24**	Root	UHPLC/ToFMS, MS	Methanol	Chen et al. (2019)
	Hyuganin C **25**	Root	UHPLC/ToFMS, MS	Methanol	Chen et al. (2019)
	Qianhucoumarin J **26**	Root	UHPLC/ToFMS, MS	Methanol	Chen et al. (2019)
	Praeruptorin B **27**	Root	UHPLC/ToFMS, MS	Methanol	Chen et al. (2019)
	(Chen et al.)-Praeruptorin A **28**	Root	HPLC, NMR	Boiling light petroleum	Xiong et al. (2012)
	Cis-3′-isovaleryl-4′-senecioylkhellactone **29**	Root	MS, SGCC, HPLC, NMR	Ethanol extract	Jong et al. (1992)
	Decursinol angelate **30**	Root	OCC, HR-TOF-MS, GPC, NMR, PHPLC, SGCC	Ethanol	Liu (2020)
	3′(S),4′(S)-3′,4′-disenecioyl-3′, 4′-dihydroseselin **31**	Root	LCC, HPLC, NMR, ACC, SGCC	Petroleum ether	Chang (1998)
	3′(R)-O-acetyl-4′(S)-O-angeloylkhellact **32**	Root	MS, PHPLC, NMR	Ethanol	Lou et al. (2004)
	3′, 4′-disenecioyl-cis-khellactone **33**	Root	AC, MS, HPLC, NMR	Crude	Cheong et al. (2002)
	Pteryxin **34**	Root	OCC, NMR, PHPLC, HR-TOF-MS, SGCC, GPC	Ethanol	Liu (2020)
	Selinidin **35**	Root	HR-ESI-MS NMR, FC/SNAP	Ethanol	Lee et al. (2015)
	Isobocconin **36**	Root	ACC, HPLC, NMR	Ethanol	Chang and Li (1999a)
	Aegelinol **37**	Root	ACC, NMR, HPLC	Ethanol	Chang and Li (1999a)
	Suksdorfin **38**	Root	FC/SNAP, NMR, HR-ESI-MS	Ethanol	Lee et al. (2015)
	D-laserpitin **39**	Root	FC/SNAP, NMR, HR-ESI-MS	Ethanol	Lee et al. (2015)
	(−)-trans-khellactone **40**	Root	EIMS, HPLC, NMR, SGCC	Petroleum ether	Kong et al. (1993b)
	(+)-cis-khellactone **41**	Root	EIMS, NMR, SGCC, HPLC	Petroleum ether	Kong et al. (1993b)
	Neopeucedalactone **42**	Root	SCC-LH20, NMR, sPHPLC, SGCC	Ethanol	Li et al. (2020)
	Decursitin D **43**	Root	NMR, SGCC, EIMS	CHCl_3_	Wang et al. (2018)
	Praeroside V **44**	Root	OHPLCc18, NMR, MS	Acetone	Takata et al. (1988)
	Cis-3′,4′-diisovalerylkhellactone **45**	Root	MS, UHPLC/ToFMS	Methanol	Chen et al. (2019)
	Praeroside III **46**	Root	NMR, OHPLCc18, MS	Acetone	Takata et al. (1988)
	Praeroside II **47**	Root	NMR, OHPLCc18, MS	Acetone	Takata et al. (1988)
	(±)-peuformosin **48**	Root	NMR, HPLC, MS	Ethanol	Chen et al. (2021)
	Praeruptorin D **49**	Root	FC/ACC, NMR	Diethyl ether/petroleum ether	Chen et al. (1979)
	Peucedanocoumarin II **50**	Root	EIMS, NMR, SSHPLC	Acetone	Takata et al. (1990)
	Isoepoxypteryxin **51**	Root	HPLC	Ethanol	Chen et al. (2021)
	Qianhucoumarin H **52**	Root	EIMS, NMR. SGCC, IR	Petrol	Kong et al. (1996)
	Praeroside IV **53**	Root	MS, NMR, OHPLCc18	Acetone	Takata et al. (1988)
	(+)-Praeruptorin A **54**	Root	NMR, HPLC, MS	Boiling light petroleum	Xiong et al. (2012)
	(±)-cis-4′-ethy-3′-tigloylkhellactone **55**	Root	NMR, OCC, HR-TOF-MS, SGCC, PHPLC, GPC	Ethanol	Liu (2020)
	(3S′,4S′)-3-angeloyloxy-4-hydroxy-3,4-dihydroSeselin **56**	Root	HR-TOF-MS, SGCC, NMR, PHPLC, OCC, GPC	Ethanol	Liu (2020)
	Hyuganin B **57**	Root	HR-TOF-MS, NMR, PHPLC, OCC, GPC, SGCC	Ethanol	Liu (2020)
	Corymbocoumarin **58**	Root	HR-TOF-MS, NMR, OCC, GPC, PHPLC, SGCC	Ethanol	Liu (2020)
	Pd-C-II **59**	Root	EIMS, NMR, PHPLC, SGCC	Ethanol	Wang et al. (2018)
	Peucedanocoumarin I **60**	Root	HR-TOF-MS, NMR, PHPLC, OCC, GPC, SGCC	Ethanol	Liu (2020)
	(+)-samidin **61**	Root	HR-TOF-MS, NMR, PHPLC, OCC, GPC, SGCC	Ethanol	Liu (2020)
	(3′S,4′S)-3′-O-isobutyroyl-4′-O-isovaleroylkhellactone **62**	Root	HR-TOF-MS, NMR, PHPLC, OCC, GPC, SGCC	Ethanol	Liu (2020)
	Pd-Ib **63**	Root	HPLC, MS	Methanol	Okuyama and Shibata (1981)
	Qianhucoumarin C **64**	Root	HR-TOF-MS, NMR, PHPLC, OCC, GPC, SGCC	Ethanol	Liu (2020)
	Pd-C-I **65**	Root	SGCC, NMR, MS	Petroleum ether	Kong et al. (1994a)
	Peucedanocoumarin III **66**	Root	SSHPLC, NMR, EIMS	Acetone	Takata et al. (1990)
Furanocoumarins	Psoralen **67**	Root, stem, leaf	HPLC-EIMS	Methanol	Jian et al. (2020)
	Angelicin **68**	Root, stem, leaf	HPLC-EIMS	Methanol	Jian et al. (2020)
	Xanthotoxin **69**	Cork, phloem, cambium, xylem, whole root	MS, HPLC-DAD	Methanol	Chen et al. (2019)
	Bergapten **70**	Cork, phloem, cambium, xylem, whole root	MS, HPLC-DAD	Methanol	Chen et al. (2019)
	Imperatorin **71**	Cork, phloem, cambium, xylem, whole root	MS, HPLC-DAD	Methanol	Chen et al. (2019)
	Deltoin **72**	Root	HR-TOF-MS, NMR, PHPLC, OCC, GPC, SGCC	Ethanol	Liu (2020)
	Isopimpinellin **73**	Root	UHPLC/ToFMS, MS	Methanol	Chen et al. (2019)
	Rutaretin **74**	Root	HPLC, NMR, MS	Ethanol	Chen et al. (2021)
	Arnocoumarin **75**	Root	MS, NMR, ACC, SGCC, HPLC	Ethanol	Chang and Li (1999b)
	Qianhucoumarin G **76**	Root	SGCC, IR, NMR, EIMS	Petrol	Kong et al. (1996)
	Nodakenetin **77**	Root	MS, NMR, UV, RLCC, MRCC, SGCC	N-butanol	Asahara et al. (1984)
	Nodakenetin tiglate **78**	Root	HPLC, 2DHCCC, NMR, ESI-MS	Ethanol	Liu et al. (2014)
	Marmesinin **79**	Root	HPLC, NMR, MS	Ethanol	Chen et al. (2021)
	Oxypeucedanin **80**	Root	SGCC, OCC, NMR, MS	Ethanol	Zhang et al. (2011)
	Marmesin-11-O-β-D-glucopyranosyl (1→6)-β-D-glucopyranoside **81**	Root	ESI-MS, NMR, HPLC	Ethanol	Wang et al. (2018)
	Rutarin **82**	Root	EIMS, NMR, HPLC	N-butanol	Okuyama et al. (1989)
	Oxypeucedanin hydrate **83**	Root	SGCC, OCC, NMR, MS	Ethanol	Zhang et al. (2011)
	Marmesin **84**	Root	HPLC, NMR, EIMS	N-butanol	Okuyama et al. (1989)
	Sphondin **85**	Root	MS, NMR, OCC, SGCC	Ethanol	Zhang et al. (2011)
	Oroselol **86**	Root	EIMS, NMR, SGP, SGCC	Ethanol	Wang et al. (2018)
	Peucedanoside A **87**	Root	MS, NMR, sPHPLC, SGCC, TLC	Methanol	Chang et al. (2007)
	Peucedanoside B **88**	Root	MS, NMR, sPHPLC, SGCC, TLC	Methanol	Chang et al. (2007)
	Apterin **89**	Root	MS, NMR, sPHPLC, SGCC, TLC	Methanol	Chang et al. (2007)
	Praeroside VII **90**	Root	NMR, TLC, sPHPLC, SGCC	Methanol	Chang et al. (2008)
	Isorutarin **91**	Root	EIMS, NMR, HPLC	N-butanol	Okuyama et al. (1989)
	Nodakenin **92**	Root	MS, UHPLC/ToFMS	Methanol	Chen et al. (2019)
	Praeroside I **93**	Root	SGCC, GPC, PHPLC, OCC, NMR, HR-TOF-MS	Ethanol	Liu (2020)
	(2′S)-rutaretin-4′-O-(6-p-hydroxybenzoyl-*β*-D-glucopyranoside) **94**	Root	MS, NMR, HPLC	Ethanol	Chen et al. (2021)
Ketone	Tanshinone I **95**	Root	MS, NMR, OCC, SGCC, MRCC	Ethanol	Zhang et al. (2005)
	Tanshinone IIA **96**	Root	MS, NMR, OCC, SGCC, MRCC	Ethanol	Zhang et al. (2005)
Sterol	β-sitosterol **97**	Stem, leaf	IR, SGCC, NMR, EIMS	Ethanol	Kong et al. (1993b)
	Daucosterol **98**	Stem, leaf	IR, SGCC, NMR, EIMS	Ethanol	Kong et al. (1993a)
Organic acid	Vanillic acid **99**	Root	SGCC, PCC, NMR, MS	Ethyl acetate	Kong et al. (1994a)
	Gallic acid **100**	Root	SGCC, PCC, NMR, MS	Ethyl acetate	Kong et al. (1994a)
	Butyric acid **101**	Root	MS, NMR, OCC, SGCC, MRCC	Ethanol	Zhang et al. (2009)
	Palmitic acid **102**	Root	MS, NMR, OCC, SGCC, MRCC	Ethanol	Zhang et al. (2006)
	4H-1-benzopyran-4-one,5-hydroxy-6-methoxy-2-phenyl-7-O-α-D-glucuronyl acid **103**	Root	MS, NMR, SGCC	Ethanol	Zhang et al. (2012)
	Tetracosanoic acid **104**	Root	MS, NMR, OCC, SGCC, MRCC	Ethanol	Zhang et al. (2006)
	9,10-dihydrophenanthrinic acid **105**	Root	UV, IR, SIMS	Ethyl acetate	Zhang et al. (2010a)
Others	2,6-dimethyl quinoline **106**	Root	MS, NMR, OCC, SGCC, MRCC	Ethanol	Zhang et al. (2006)
	3-(4′-for mylphenoxy)-4-methoxybenzaldehyde **107**	Root	HPLC, NMR, MS	Ethanol	Chen et al. (2021)
	3-(4′-formylphenoxy)-4-methoxybenzaldehyde **108**	Root	HR-TOF-MS, NMR, PHPLC, OCC, GPC, SGCC	Ethanol	Liu (2020)
	Bis(2-ethylhexyl) phthalate **109**	Root	HR-TOF-MS, NMR, PHPLC, OCC, GPC, SGCC	Ethanol	Liu (2020)
	4-[β-D-apiofuranosyl-(1→6)-β-D-glucopyranosyloxy]-3-methox ypropiophenone **110**	Root	HPLC, NMR, MS	Ethanol	Chen et al. (2021)
	Baihuaqianhuoside **111**	Root	MS, NMR, PHPLC, SGCC	Ethanol	Asahara et al. (1984)
	Galactitol **112**	Root	EIMS, NMR, SGCC	Ethanol	Kong et al. (1993a)
	(−)-sclerodin **113**	Root	MS, NMR, OCC, SGCC, MRCC	Ethanol	Zhang et al. (2006)
	Adenoside **114**	Root	MRCC, SGCC, OCC, NMR, MS	Ethanol	Zhang et al. (2009)
	Acetylatractylodinol **115**	Root	MS, NMR, OCC, SGCC, MRCC	Ethanol	Zhang et al. (2005)
	4H-1-benzopyran-4-one,5-hydroxy-6-methoxy-2-phenyl-7-O-α-D-glucuronyl methyl ester **116**	Root	MS, NMR, OCC, SGCC	Ethanol	Zhang et al. (2012)
	Polyacetylene **117**	Root	CS, NMR, HR-ESI-MS	Ethanol	Lee et al. (2015)
	D-mannitol monohexadecanoate **118**	Root	MS, NMR, OCC, SGCC, MRCC	Ethanol	Zhang et al. (2009)
	α-D-glucopyranose-1-hexadecanoate **119**	Root	MS, NMR, OCC, SGCC, MRCC	Ethanol	Zhang et al. (2009)

^a^ Note: adsorption chromatography (AC); alumina column chromatography (ACC); chromatographic separation (CS); electrospray ionization mass spectrometry (ESI-MS); flash chromatography using SNAP, Ultra cartridge (FC/SNAP); fractional crystallization/alumina column chromatography (FC/ACC); gel permeation chromatography (GPC); high performance liquid chromatography with octadecylsilyl (c18) (OHPLCc18); high performance liquid chromatography (HPLC); high performance liquid chromatography with octadecylsilyl (c18)/develosil (c30) (OHPLCc18/c30); high performance liquid chromatography-diode-array detector (HPLC-DAD); high performance liquid chromatography-electrospray ionization mass spectrometry (HPLC-EIMS); high-resolution electron ionization mass spectrometry (HREIMS); high-resolution-electrospray ionization-mass spectrometry (HR-ESI-MS); high-resolution-time-of-flight mass spectrometry (HR-TOF-MS); infrared spectroscopy (IR); lobar column chromatography (LCC); macroporous resin column chromatography (MRCC); mass spectrometry (MS); nuclear magnetic resonance (NMR); octadecylsilyl column chromatography (OCC); polyphthalamide column chromatography (PCC); preparative high performance liquid chromatography (PHPLC); Rp-8, reversed lobar column chromatography (RLCC); secondary ion mass spectroscopy (SIMS); semi-preparative high performance liquid chromatography (sPHPLC); Senshu scientific high performance liquid chromatography (SSHPLC); Sephadex gel purification (SGP); Sephadex LH-20, column chromatography (SCC-LH20); silica gel column chromatography (SGCC); thin-layer chromatography (TLC); two-dimensional hyphenation of counter-current chromatography (2DHCCC); ultra-high performance liquid chromatography/time of flight mass spectrometry (UHPLC/ToFMS); ultraviolet spectrum (UV).

### 5.1 Simple coumarins

**FIGURE 3:**
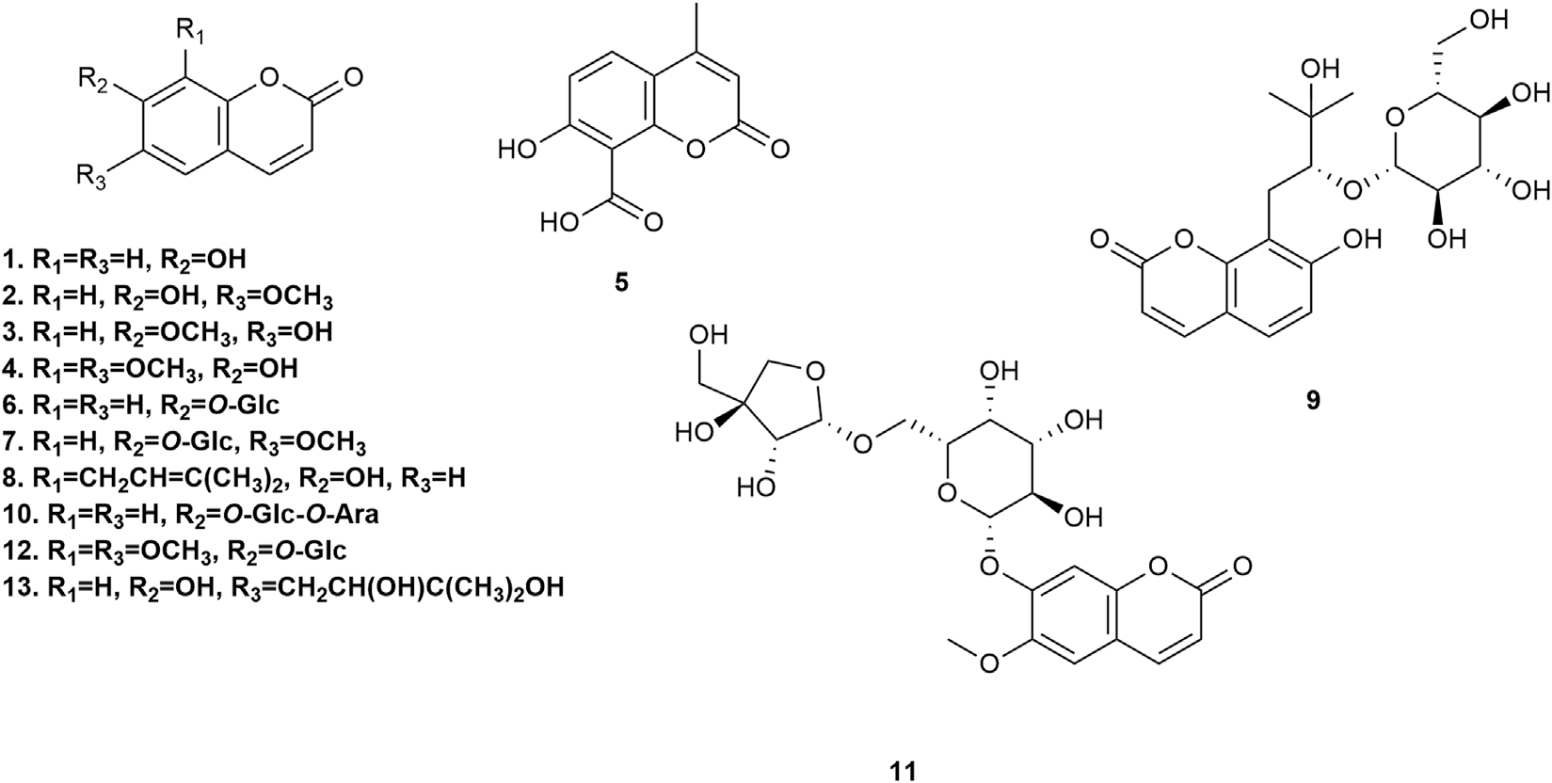
Simple coumarins isolated from *Peucedanum praeruptorum* Dunn.

Thirteen simple coumarins (Figure 3) have been isolated from *P. praeruptorum* root tissues, including umbelliferone **1**, scopoletin **2**, isoscopoletin **3**, isofraxidin **4**, 8-carboxy-7-hydroxy coumarin **5**, skimmin **6**, scopolin **7**, osthenol **8**, praeroside VI **9**, apiosylskimmin **10**, hymexelsin **11**, eleutheroside B1 **12**, and (−)-peucedanol **13**. However, the pharmacological activities of these simple coumarins have rarely been reported.

### 5.2 Pyranocoumarins

**FIGURE 4:**
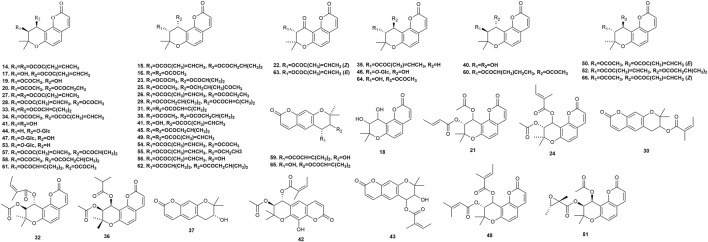
Pyranocoumarins isolated from *Peucedanum praeruptorum* Dunn.

Fifty-five pyranocoumarins (Figure 4) have been isolated from *P. praeruptorum* root tissues, including praeruptorin C **14**, praeruptorin E **15**, qianhucoumarin D **16**, qianhucoumarin A **17**, khellactone **18**, qianhucoumarin B **19**, (9R,10R)-9-acetoxy-8,8-dimethyl-9,10-dihydro-2H, 8H-benzo [1,2-b:3,4-b′]dipyran-2-one-10-yl-ester **20**, (±)-cis-4′-acetyl-3′-crotonoykhellactone **21**, qianhucoumarin E **22**, hyuganin D **23**, qianhucoumarin I **24**, hyuganin C **25**, qianhucoumarin J **26**, praeruptorin B **27**, (−)-praeruptorin A **28**, cis-3′-isovaleryl-4′-senecioylkhellactone **29**, decursinol angelate **30**, 3′(S),4′(S)-3′,4′-disenecioyl-3′,4′-dihydroseselin **31**, 3′-O-acetyl-4′(S)-O-angeloyl-khellact **32**, 3′,4′-disenecioyl-cis-khellactone **33**, pteryxin **34**, selinidin **35**, isobocconin **36**, aegelinol **37**, suksdorfin **38**, D-laserpitin **39**, (−)-trans-khellactone **40**, (+)-cis-khellactone **41**, neopeucedalactone **42**, decursitin D **43**, praeroside V **44**, cis-3′,4′-diisovalerylkhellactone **45**, praeroside III **46**, praeroside II **47**, (±)-peuformosin **48**, praeruptorin D **49**, peucedanocoumarin II **50**, isoepoxypteryxin **51**, qianhucoumarin H **52**, praeroside IV **53**, (+)-praeruptorin A **54**, (±)-cis-4′-ethy-3′-tigloylkhellactone **55**, (3S′,4S′)-3-angeloyloxy-4-hydroxy-3,4-dihydroseselin **56**, hyuganin B **57**, corymbocoumarin **58**, Pd-C-II **59**, peucedanocoumarin I **60**, (+)-samidin **61**, (3′S, 4′S)-3′-O-isobutyroyl-4′-O-isovaleroylkhellactone **62**, Pd-Ib **63**, qianhucoumarin C **64**, Pd-C-I **65**, and peucedanocoumarin III **66**. Research suggests that praeruptorin B possesses antitumor activity, and praeruptorin E possesses anti-inflammatory activity (Yu et al., 2012; Lin et al., 2020).

### 5.3 Furanocoumarins

**FIGURE 5:**
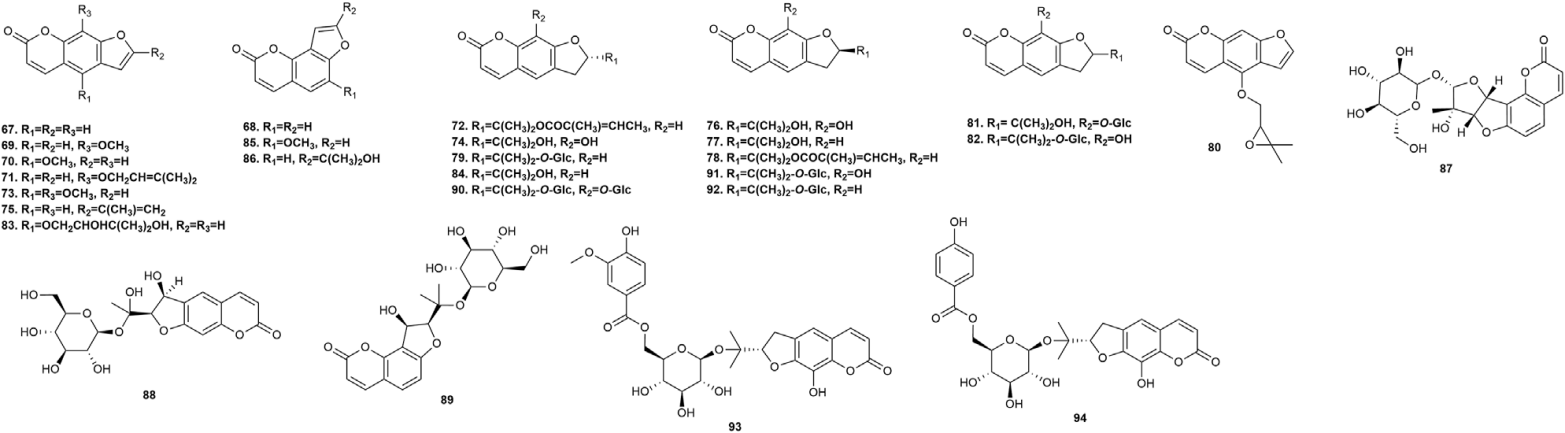
Furanocoumarins isolated from *Peucedanum praeruptorum* Dunn.

Furanocoumarins possess neuroprotective, anti-inflammatory, and anticancer activities in animals, and serve as phytotoxins and allelochemicals in plants (Jian et al., 2020). Twenty-nine furanocoumarins (Figure 5) have been isolated from *P. praeruptorum* root tissues, including psoralen **67**, angelicin **68**, xanthotoxin **69**, bergapten **70**, imperatorin (IMP) **71**, deltoin **72**, isopimpinellin **73**, rutaretin **74**, arnocoumarin **75**, qianhucoumarin G **76**, nodakenetin **77**, nodakenetin tiglate **78**, marmesinin **79**, oxypeucedanin **80**, marmesin-11-O-β-D-glucopyranosyl (1→6)-β-D-glucopyranoside **81**, rutarin **82**, oxypeucedanin hydrate **83**, marmesin **84**, sphondin **85**, oroselol **86**, peucedanoside A **87**, peucedanoside B **88**, apterin **89**, praeroside VII **90**, isorutarin **91**, nodakenin **92**, praeroside I **93**, and (2′S)-rutaretin-4′-O-(6-p-hydroxybenzoyl-β-D-glucopyranoside) **94.**

### 5.4 Ketones, sterols, and organic acids

**FIGURE 6:**
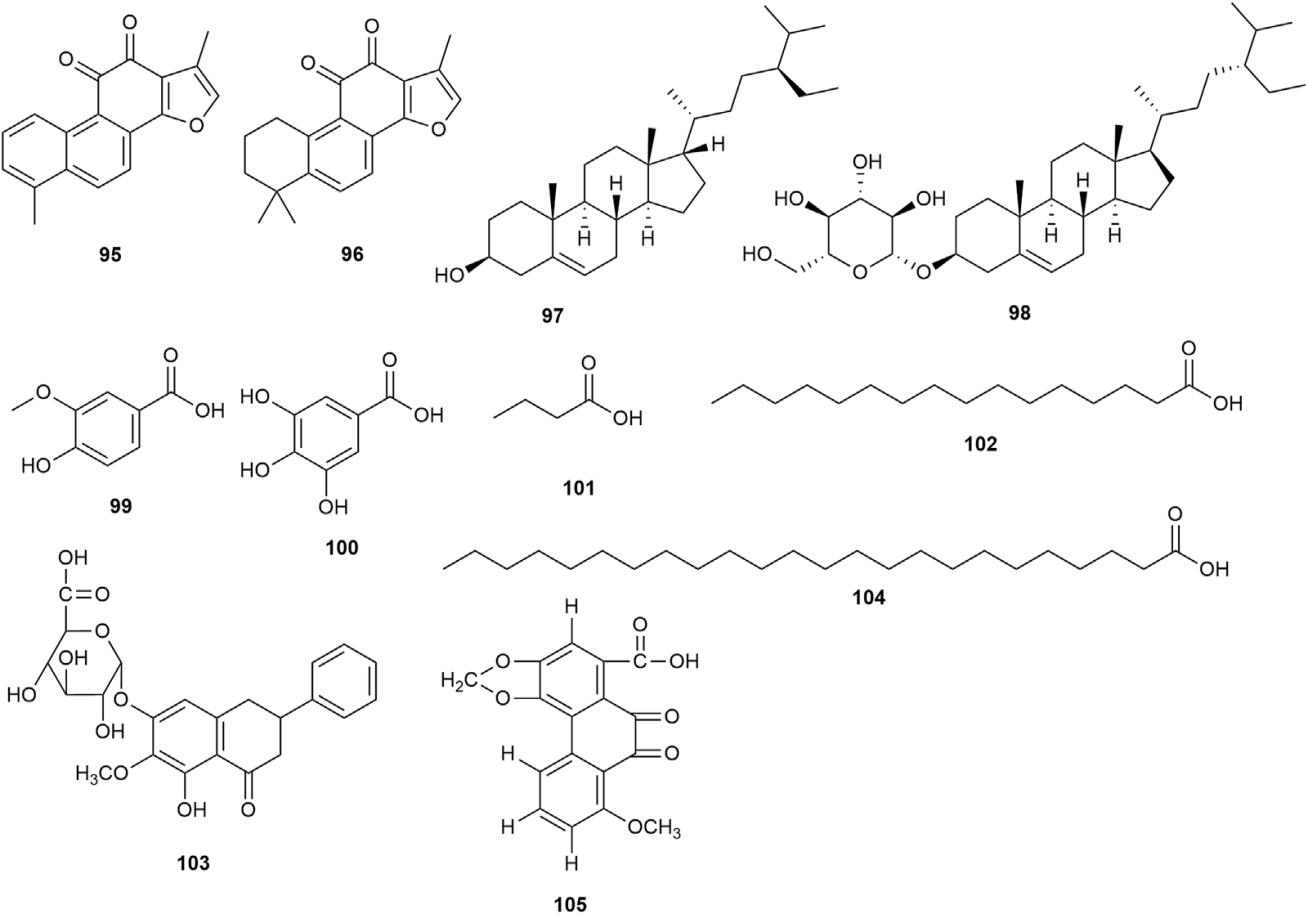
Ketones, sterols, and organic acids isolated from *Peucedanum praeruptorum* Dunn.

Two ketones (tanshinone I **95** and tanshinone IIA **96**) were confirmed in the roots of *P. praeruptorum*. Two sterols (β-sitosterol **97** and daucosterol **98**) and seven organic acids (vanillic acid **99**, gallic acid **100**, butyric acid **101**, palmitic acid **102**, 4H-1-benzopyran-4-one,5-hydroxy-6-methoxy-2-phenyl-7-O-α-D-glucuronyl acid **103**, tetracosanoic acid **104**, and 9,10-dihydrophenanthrinic acid **105**) were confirmed in the stem and leaves of *P. praeruptorum*. However, the pharmacological activities of these ketones, sterols, and organic acids were not found in the available studies. Figure 6 shows the chemical structures of these phytochemicals.

### 5.5 Other metabolites

**FIGURE 7:**
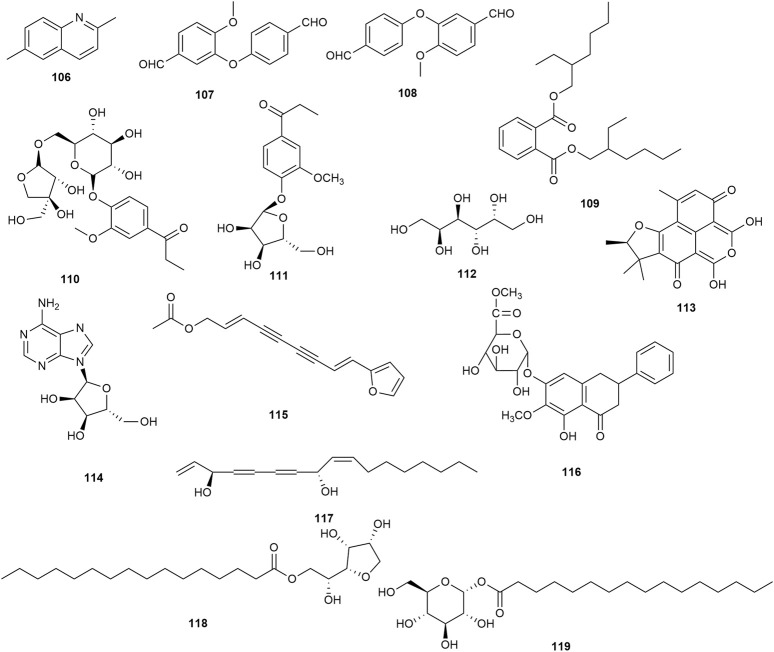
Additional phytochemicals isolated from *Peucedanum praeruptorum* Dunn.

Other metabolites (Figure 7), such as 2,6-dimethyl quinoline **106**, 3-(4′-formylphenoxy)-4-methoxybenzaldehyde **107**, 3-(4′-formylphenoxy)-4-methoxybenzaldehyde **108**, bis(2-ethylhexyl) phthalate **109,** 4-[β-D-apiofuranosyl-(1→6)-β-D-glucopyranosyloxy]-3-methoxypropiophenone **110**, baihuaqianhuoside **111**, galactitol **112**, (−)-sclerodin **113**, adenoside **114**, acetylatractylodinol **115**, 4H-1-benzopyran-4-one,5-hydroxy-6-methoxy-2-phenyl-7-O-α-D-glucuronyl methyl ester **116**, polyacetylene **117**, D-mannitol monohexadecanoate **118**, and α-D-glucopyranose-1-hexadecanoate **119**, have been isolated from *P. praeruptorum* root tissues. However, the pharmacological activities of these phytochemicals were not found in the available studies.

## 6 Pharmacological activities

**FIGURE 8:**
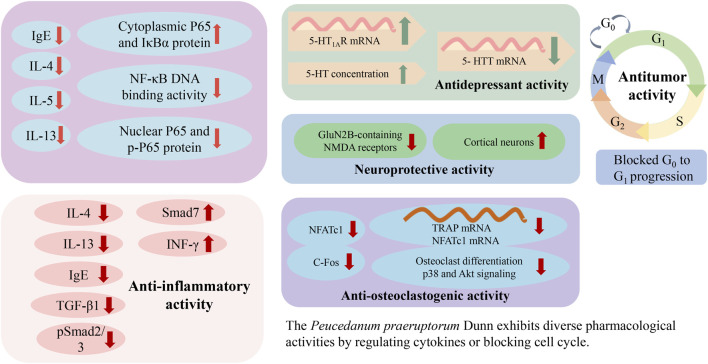
Possible mechanisms associated with the pharmacology of *Peucedanum praeruptorum* Dunn.

*P. praeruptorum* exhibits diverse pharmacological activities (Table 3), including anti-inflammatory, expectorant, antitussive, antitumor, neuroprotective, anti-osteoclastogenic, and antidepressant effects. The antitumor, immunoregulatory, and anti-inflammatory activities are the most notable, and putative molecular mechanisms are shown in Figure 8.

**TABLE 3:** Pharmacology of phytochemicals extracted from *Peucedanum praeruptorum* Dunn.

Pharmacological activity	Tested substance	Model	Experimental system	Type of study	Results	Dose range	Application period	References
Anti-inflammatory activity	Praeruptorin C	Mouse	Left paw	*In vivo*	Inhibited microglial activation; attenuated proinflammatory cytokine release; regulated excitatory transmission in ACC of CFA-injected mice	3 mg/kg	21 d	Su et al. (2021)
	(±)-praeruptorin A	Murine model of chronic asthma	Lung	*In vivo*	Decreased expression of IgE (serum) and IL-4/-13 (BALF); suppressed airway inflammation, hyperresponsiveness, and remodeling; constrained TGF-β1 and pSmad2/3 expression and promoted Smad7 expression (lung tissue) and INF-γ (BALF)	30, 60, 120 mg/kg	56 d	Xiong et al. (2012)
	Praeruptorin D	Inflammatory Periodontal membrane cells	Cell culture	*In vitro*	Constrained TNF-ɑ and IL-1β expression	10, 20, 30, 40 μg/mL	1, 2, 3, 5 d	Yu (2022)
	Praeruptorin C	LPS-stimulated raw264.7 macrophage cells	Cell culture	*In vitro*	Inhibited NF-κB and STAT3 activation	2, 4, 8, 16 μg/mL	18 h	Yu et al. (2012)
	Praeruptorin D	LPS-stimulated raw264.7 macrophage cells	Cell culture	*In vitro*	Inhibited NF-κB and STAT3 activation	2, 4, 8, 16 μg/mL	18 h	Yu et al. (2012)
	Praeruptorin E	LPS-stimulated raw264.7 macrophage cells	Cell culture	*In vitro*	Inhibited NF-κB and STAT3 activation	2, 4, 8, 16 μg/mL	18 h	Yu et al. (2012)
	Imperatorin	RBL-2H3 allergic inflammatory cell	Cell culture	*In vitro*	Inhibited the degranulation rate of RBL-2H3 cells; inhibited the release of histamine, IL-3/-4/-6, TNF-α, and COX-2; promoted the expression of IFN-γ	5, 10, 15 μmol/L	1 h	Long et al. (2019)
	*P. praeruptorum* polysaccharides	RAW264.7 macrophages	Cell culture	*In vitro*	Increased accessory and costimulatory molecule expression, the secretion of chemokines and inflammatory factors, and phagocytosis/pinocytosis	25, 50, 100, 200 μg/mL	24 h	Zhao et al. (2022)
	Dl- praeruptorin A	LPS mouse model of acute lung injury	Lung	*In vivo*	Reduced lung inflammation	10 μg/g	24 h	Zhou et al. (2016)
	Dl- praeruptorin A	LPS-induced HUVECs	Cell culture	*In vitro*	Inhibited LPS-induced endothelial inflammation	10, 20, 40 μmol/L	24 h	Wang et al. (2012)
Expectorant and antitussive effects	*Peucedanum praeruptorum* Dunn water extract	Mouse	Trachea	*In vivo*	Reduced phlegm	45 g/kg	1 h	Liu et al. (1997), Meng et al. (1997)
	Praeruptorin C	Mouse	Trachea	*In vivo*	Reduced phlegm	3 mg/kg, 10 mg/kg	1 h	Liu et al. (2009)
	Nodakenin	Mouse	Trachea	*In vivo*	Reduced phlegm	3 mg/kg, 10 mg/kg	1 h	Liu et al. (2009)
	Nodakenin	BALB/c mouse	Mouse	*In vivo*	Decreased expression of IgE (serum) and IL-4/-13/-5 (BALF); suppressed airway inflammation and hyperresponsiveness; constrained nuclear P65/p-P65; promoted cytoplasmic P65 and IκBα; promoted DNA binding activity of NF-κB	10 mg/kg	5 d	Xiong et al. (2014)
	Raw Qianhu	Mouse	Trachea	*In vivo*	Strong expectorant and antitussive effects	2.5, 5.0, 10.0 g/kg	6 d	Zhang et al. (2010b)
	Honey-roasted Qianhu	Mouse	Trachea	*In vivo*	Strong expectorant and antitussive effects	2.5, 5.0, 10.0 g/kg	6 d	Zhang et al. (2010b)
	Raw Qianhu	Guinea pigs	Guinea pig	*In vivo*	Asthma relief	2, 4, 8 g/kg	3 d	Zhang et al. (2010b)
	Honey-roasted Qianhu	Guinea pigs	Guinea pig	*In vivo*	Asthma relief	2, 4, 8 g/kg	3 d	Zhang et al. (2010b)
Antitumor activity	Praeruptorin B	Human RCC cell lines 786-O and ACHN	Cell culture	*In vitro*	Inhibited migrability and invasibility; inhibited cathepsin C and cathepsin V expression in ACHN and 786-O cell lines	0, 10, 20, 30 μmol/L	24 h	Lin et al. (2020)
	Praeruptorin B	Ovarian cancer SK-OV-3 cells	Cell culture	*In vitro*	Inhibited SK-OV-3 cellular proliferation and migration; reduced the expression of c-myc, cyclind1, srebp-1c, and fasn mRNA/protein	20, 40, 60 μmol/L	24 h	Xue et al. (2021)
	Praeruptorin A	Ovarian cancer A2780/TAX cells	Cell culture	*In vitro*	Inhibited A2780/TAX cellular proliferation, viability, and migration via promoting apoptosis	25 μmol/L	48 h	Chen et al. (2022)
	Praeruptorin A	HeLa cells	Cell culture	*In vitro*	Induction of G0/G1 phase cell cycle arrest; upregulated expression of tissue inhibitor of metalloproteinase-2, Rb, and p16/21/27; downregulated expression of matrix metalloproteinase-2, S-phase kinase-associated protein 2, and cyclin D1	0, 10, 20, 30 μmol/L	24 h	Wu et al. (2017)
	Praeruptorin A	HeLa cells	Cell culture	*In vitro*	Enhanced the ability of MEK1/2 inhibitor PD98059 to downregulate metalloproteinase-2; suppressed the activation of SERK1/2; upregulated expression of tissue inhibitor of metalloproteinase-2	0, 20 μmol/L	24 h	Wu et al. (2017)
	Praeruptorin A	SiHa cells	Cell culture	*In vitro*	Upregulated the expression of tissue inhibitor of metalloproteinase-2; downregulated the expression of matrix metalloproteinase-2	0, 10, 20, 30 μmol/L	24 h	Wu et al. (2017)
	Praeruptorin A	Human HCC cells	Cell culture	*In vitro*	Reduced the migrability and invasibility of human HCC cells; activated extracellular signal-regulated kinase signaling; downregulated matrix metalloproteinase-1 expression	0, 10, 20, 30 μmol/L	24 h	Yu et al. (2021)
	Praeruptorin A	LS174T cells	Cell culture	*In vitro*	Pregnane X receptor-mediated induction of cytochrome P450 3A4 expression and activity	2.5, 10, 40 μmol/L	48 h	Huang et al. (2013)
	Praeruptorin A	SGC7901 human gastric cancer cells	Cell culture	*In vitro*	Cytotoxicity toward SGC7901 cells	10, 50, 100 μmol/L	24 h	Liang et al. (2010)
	Praeruptorin B	SGC7901 human gastric cancer cells	Cell culture	*In vitro*	Cytotoxicity toward SGC7901 cells	10, 50, 100 μmol/L	24 h	Liang et al. (2010)
	Praeruptorin A	SGC7901 human gastric cancer cells	Cell culture	*In vitro*	Complemented the effect of Doxorubincin on SGC7901 cells	50, 100 μmol/L	24 h	Liang et al. (2010)
	Praeruptorin A	HepG2 cells	Cell culture	*In vitro*	Constitutive androstane receptor-mediated upregulation of multidrug resistance-associated protein 2 *in vitro*	10, 25, 50 μmol/L	24 or 28 h	Zhou et al. (2013)
	Praeruptorin C	HepG2 cells	Cell culture	*In vitro*	Constitutive androstane receptor-mediated upregulation of multidrug resistance-associated protein 2 *in vitro*	10, 25, 50 μmol/L	24 or 28 h	Zhou et al. (2013)
	Praeruptorin A	H1975 (EGFR L858R/T790M double-mutant, EGFR TKI-resistant) human non-small-cell lung cancer cells	Cell culture	*In vitro*	Induced apoptosis in H1975 cells	0, 50, 100 μg/mL	72 h	Park et al. (2022)
	Pteryxin	H1975 (EGFR L858R/T790M double-mutant, EGFR TKI-resistant) human non-small-cell lung cancer cells	Cell culture	*In vitro*	Induced apoptosis in H1975 cells	0, 50, 100 μg/mL	72 h	Park et al. (2022)
	Praeruptorin A	H1975 (EGFR L858R/T790M double-mutant, EGFR TKI-resistant) human non-small-cell lung cancer cells, PC9/ER (erlotinib-resistant) human non-small-cell lung cancer cells	Cell culture	*In vitro*	Suppressed HGF-induced phosphorylation of MET.	0, 50, 100 μg/mL	2 h	Park et al. (2022)
	Pteryxin	H1975 (EGFR L858R/T790M double-mutant, EGFR TKI-resistant) human non-small-cell lung cancer cells, PC9/ER human non-small-cell lung cancer cells	Cell culture	*In vitro*	Suppressed HGF-induced phosphorylation of MET.	0, 50, 100 μg/mL	2 h	Park et al. (2022)
	Praeruptorin A	H1975 (EGFR L858R/T790M double-mutant, EGFR TKI-resistant) human non-small-cell lung cancer cells	Cell culture	*In vitro*	Dephosphorylated AKT.	0, 50, 100 μg/mL	2 h	Park et al. (2022)
	(±)-4′-O- acetyl-3′-O-angeloyl- cis- khellactone	U266 cells	Cell culture	*In vitro*	Induced apoptosis to suppress cell proliferation	0, 10, 20, 30, 40 μg/mL	24 h	Yu et al. (2015)
	Neopeuceda-lactone	Human leukemic HL-60 cell lines	Cell culture	*In vitro*	Inhibited cell growth *in vitro*	-	3 d	Li et al. (2020)
	Neopeuceda-lactone	Human leukemic THP-1 cell lines	Cell culture	*In vitro*	Inhibited cell growth *in vitro*	-	3 d	Li et al. (2020)
	Neopeuceda-lactone	Human prostate cancer PC-3 cell lines	Cell culture	*In vitro*	Inhibited cell growth *in vitro*	-	3 d	Li et al. (2020)
Neuroprotective activity	Praeruptorin C	Primary neurons	Cell culture	*In vitro*	Reversed N-methyl-D-aspartate-induced upregulation of GluN2B-containing N-methyl-D-aspartate receptors	0, 1, 10 μmol/L	24 h	Yang et al. (2013)
	Praeruptorin C	Primary neurons	Cell culture	*In vitro*	Inhibited N-methyl-D-aspartate-induced neuronal apoptosis via reversing intracellular Ca^2+^ overload and balancing the Bcl-2/Bax ratio	0, 1, 10 μmol/L	24 h	Yang et al. (2013)
	Praeruptorin C	3-nitropropionic-treated acid mouse	Mouse	*In vivo*	Alleviated excitotoxicity, motor deficits, and depressive behavior in 3-nitropropionic acid-treated mice	1.5, 3.0 mg/kg	3 d	Wang et al. (2017)
Anti-osteoclastogenic activity	Praeruptorin C	Osteoclasts	Cell culture	*In vitro*	Attenuated the formation of osteoclasts via inhibition of JNK and NF-κB pathways, without altering p38 or ERK.	0, 20 μmol/L	4 h	Liu et al. (2017)
	Praeruptorin C	OVX mouse	Mouse	*In vivo*	Constrained osteoclastic bone resorption and F-actin ring formation	5, 10 μmol/L	28 d	Liu et al. (2017)
	Praeruptorin A	Bone marrow–derived macrophages	Cell culture	*In vitro*	Constrained Akt and p 38 signaling, and RANKL-mediated osteoclast differentiation	10 μmol/L	30 min	Yeon et al. (2014)
	*Peucedanum praeruptorum* Dunn	Inflammatory periodontal membrane cells	Cell culture	*In vitro*	Induced the expression of osteogenic genes RUNX-2, ALP, and OCN, and differentiation of inflammatory periodontal membrane cells	10, 20, 30, 40 μg/mL	1, 2, 3, 5 d	Yu (2022)
Antidepressant activity	Dl- praeruptorin A	Chronic unpredicted mildly stressed rat	Rat	*In vivo*	Improved the depressive behavior of chronic mildly-stressed rats	10, 30, 60 mg/kg	28 d	Wang and Xu (2014)
	Imperatorin	Rat (male offspring)	Rat	*In vivo*	Enhanced 5-HT_1A_R expression, 5-HT level, and sucrose preference; increased the incidence of grooming, rearing, and crossing behaviors; reduced immobility; and decreased 5-HTT expression	15, 30 mg/kg	28 d	Zheng et al. (2019)
Other activity	*P. praeruptorum* alcohol extracts	Rat	Left ventrical	*In vivo*	Affected ventricular remodeling and apoptosis-related proteins in different ways, and had a positive influence on ventricular remodeling	1, 2, 4 g/mL	28 d	Tu et al. (2004)
	Total courmarins	Mouse	Mouse	*In vivo*	Prolonged the hypnotic duration of pentobarbital sodium in a dose-dependent manner; inhibited the activities of aniline hydroxylase and aminopyrine N-demethylase; minimally influenced the hypnotic effect of barbital sodium; inhibited the activity of hepatic microsomal drug-metabolizing enzymes	50, 100, 200 mg/kg	1 h	Wang et al. (2004)
	Nodakenin	Mouse	Mouse	*In vivo*	Reversed scopolamine-induced cognitive impairments	0, 2.5, 5, 10, 20 mg/kg	4 d	Kim et al. (2007)
	Nodakenin	Mouse	Mouse	*In vivo*	Inhibited acetylcholinesterase activity	10 mg/kg	24 h	Kim et al. (2007)
	Dl-praeruptorin A	Rat	Cell culture	*In vitro*	Prevented postischemic cell death in rat heart	0.5, 1.0, 2.0 mg/kg	2 h	Chang et al. (2003)
	Praeruptorin C	Rat	Myocardial tissue	*In vitro*	Reduced ischemia/reperfusion injury induced by coronary ligation	5, 15, 30 mg/kg	3 d	Liu and Wang (2009)

“-” denotes no useful information found in the study.

### 6.1 Anti-inflammatory activity

Praeruptorin C (pyranocoumarins) was traditionally used as an antibechic and antibronchitic drug. In one study, praeruptorin C treatment was found to regulate excitatory synaptic proteins in the anterior cingulate cortex, attenuate the release of proinflammatory cytokines, and inhibit the activation of microglia (Su et al., 2021). However, further studies need to assess the effects of praeruptorin C in other pain models. In another study, administration of (±)-praeruptorin A (30, 60, 120 mg/kg) to ovalbumin-sensitized BALB/c mice for 56 days increased the level of INF-γ and reduced the expression of IL-13/-4 in bronchoalveolar lavage fluid (BALF); decreased the level of immunoglobulin (Ig) E in serum; and suppressed airway inflammation, hyperresponsiveness, and remodeling. In the study, levels of cytokines in BALF, immunoglobulin (Ig) E in serum as well as expression of TGF-β1 and Smad proteins in lung tissue were measured by enzyme-linked immunosorbent assay, immunohistochemistry or Western blot analysis. (Xiong et al., 2012). Treatment of inflamed human periodontal membrane cells with praeruptorin D (10, 20, 30, 40 μg/mL) inhibited TNF-α and IL-1β expression. According to experimental requirements, the negative control group was healthy cells group and positive control group was minocycline hydrochloride group (Yu, 2022). Administration of praeruptorins C, D, and E (2, 4, 8, 16 μg/mL) to RAW264.7 macrophages stimulated with lipopolysaccharide (LPS) for 24 h inhibited NF-κB and STAT3 activation. Although all three had anti-inflammatory activities, praeruptorins D and E exhibited greater anti-inflammatory activities than praeruptorin C and *in vivo* pharmacological potencies need to be further evaluated. (Yu et al., 2012). One study demonstrated that the administration of IMP (furanocoumarins) at doses of 5, 10, and 15 μmol/L for 1 h in the RBL-2H3 allergic inflammatory cell model promoted IFN-γ expression; decreased the expression of TNF-α, COX-2, IL-6, IL-4, and IL-3; and inhibited RBL-2H3 cell degranulation. These results indicate that IMP is effective for inhibiting the inflammatory response in the RBL-2H3 allergic inflammatory cell model mediated by IgE immunoregulation (Long et al., 2019). However, the authors did not evaluate the effectiveness of IMP using animal models. Moreover, *P. praeruptorum* polysaccharide treatments ranging from 25 to 200 μg/mL increased the expression of costimulatory and accessory factors, increased the secretion of chemokines and inflammatory factors, and enhanced phagocytosis and pinocytosis. In this way, *P. praeruptorum* polysaccharides modulated the inflammatory response of macrophages via the NF-κB and TLR2/TLR4-dependent MAPK pathways (Zhao et al., 2022). Another study demonstrated that Dl-praeruptorin A reduced inflammation in an LPS-induced acute lung injury mouse model, specifically inhibiting endothelial inflammation (Wang et al., 2012; Zhou et al., 2016). Nonetheless, the mechanism of Dl-praeruptorin A against acute lung injury needs further study prior to its use in clinical treatment.

### 6.2 Expectorant and antitussive effects

*P. praeruptorum* water extract treatment (45 g/kg) was found to resolve phlegm (Liu et al., 1997; Meng et al., 1997). The administration of praeruptorin C and nodakenin (10 mg/kg) to mice, with phenolsulfonphthalein as an expectorant indicator, increased phenolsulfonphthalein excretion in tracheal tissues. Both praeruptorin C and nodakenin showed expectorant effects. And ammonium chloride served as a positive control (Liu et al., 2009). However, phenolsulfonphthalein is mainly excreted from the urine by the kidneys, and drugs that affect renal function are prone to false positive results. Similarly, treating BALB/c mice for 5 days with nodakenin (10 mg/kg) promoted the DNA binding activity of NF-κB, increased the levels of IκBα and P65 in the cytoplasm, decreased the expression of p-P65 and P65 in the nucleus, reduced the level of IgE in serum and IL-13/-5/-4 in BALF, and suppressed airway hyperreactivity and inflammation (Xiong et al., 2014). Notably, the pharmacological properties of *P. praeruptorum* before and after honey roasting were found to be different. The 5.0 and 10.0 g/kg doses of honey-roasted products showed stronger expectorant and antitussive effects than raw products. However, the 2.5 g/kg dose of honey-roasted and raw products was more effective as relieving asthma (Zhang et al., 2010b). Although *P. praeruptorum* has long been used to resolve phlegm, descend Qi, clear heat, and dissipate wind in TCM (Zhao et al., 2022), comprehensive studies of its constitutive bioactive monomers and their molecular mechanisms, as well as clinical trials, are necessary to improve its expectorant and antitussive activities with minimal side effects.

### 6.3 Antitumor activity

More than 85% of all kidney cancers worldwide are characterized as renal cell carcinoma (RCC). In one study, treating RCC cells for 24 h with praeruptorin B (0–30 μmol/L) inhibited both migrability and invasibility, as well as downregulated the expression of cathepsins V and C in ACHN and 786-O cells (Lin et al., 2020). Similarly, praeruptorin B (20, 40, 60 μmol/L) was reported to inhibit both the proliferation and migration of SK-OV-3 ovarian cancer cells, as well as downregulate the expression of FASN, c-Myc, SREBP-1c, and cyclin D1. The likely mechanism is that the SREBP-1c/FASN signaling pathway regulates the energy metabolism of SK-OV-3 ovarian cancer cells, thus inhibiting their proliferation. Real-time fluorescence quantitative polymerase chain reaction (RT-qPCR) was used to detect the mRNA expressions of proliferating genes such as c-Myc and CyclinD1 and the mRNA expressions of key genes of energy metabolism such as SREBP-1c and FASN in tumor cells and Western blot was used to detect the expressions of SREBP-1c, FASN proteins (Xue et al., 2021).

A 48 h treatment with praeruptorin A (25 μmol/L) combined with taxol (125 nmol/L) induced apoptosis in A2780/TAX ovarian cancer cells, and inhibited their migration by downregulating MMP9 and MMP2 expression. However, it remains to be seen whether praeruptorin A can enhance the chemotherapeutic efficacy of taxol, or whether it retains its curative effect against ovarian cancer *in vivo* (Chen et al., 2022). Another study on HeLa and SiHa cell lines reported that praeruptorin A could increase the levels of tissue inhibitors of metalloproteinase-2 and decrease the expression of matrix metalloproteinase-2; downregulate S-phase kinase-associated protein 2 and cyclin D1; upregulate p27, p21, p16, and Rb; and induce G_0_/G_1_ phase cell cycle arrest. However, praeruptorin A could not inhibit cell viability in IgG-treated cells, the effect of IgG interference with praeruptorin A in HeLa cells (Wu et al., 2017). Additionally, praeruptorin A has been found effective at inhibiting the migrability and invasibility of hepatocellular carcinoma (HCC) cells by activating extracellular signal-regulated kinase signaling and inhibiting matrix metalloproteinase-1 expression. However, *in vivo* metastasis animal model is even worthier for further investigation to examine the antimetastatic effect and safety evaluation of praeruptorin A (Yu et al., 2021). In LS174T cells, praeruptorin A (2.5, 10, 40 μmol/L) could significantly upregulate cytochrome P450 3A4 levels and activity via a pregnane X receptor-mediated pathway. However, siRNA knockdown of the pregnane X receptor resulted in suppressed expression of cytochrome P450 3a11 in mouse primary hepatocytes (Huang et al., 2013).

Administration of praeruptorins A and B to SGC7901 human gastric cancer cells for 24 h produced cytotoxic effects in SGC7901 cells, resulting in antiproliferation. praeruptorin A could also enhance the action of doxorubicin on SGC7901 cells (Liang et al., 2010). Administration of praeruptorins A and C (10, 25, 50 μmol/L) to HepG2 cells *in vitro* for 24 or 48 h increased the expression of multidrug resistance-associated protein 2 by way of the constitutive androstane receptor-mediated pathway (Zhou et al., 2013). In H1299, PC9, H1975, and PC9/ER human non-small-cell lung cancer (NSCLC) cell lines, praeruptorin A and pteryxin restricted the HGF-induced phosphorylation of MET in PC9/ER and H1975 cells, increased PARP cleavage in H1975 cells and the proportion of annexin V-positive cells, and overall induced apoptosis and reduced cell viability. However, praeruptorin A and pteryxin could not inhibit HGF-induced AKT phosphorylation and prompted apoptosis in NSCLC cells regardless of EGFR TKI resistance or epidermal growth factor receptor (EGFR) mutation status (Park et al., 2022).

In another study, 24 h of treatment with the angular pyranocoumarin (±)-4′-O-acetyl-3′-O-angeloyl-cis-khellactone (0, 10, 20, 30, 40 μg/mL) was found to promote apoptosis in U266 cells, thereby constraining proliferation. The most likely mechanism involved the upregulation of caspase-3/-8 expression and the downregulation of hTERT, p-AKT, and pERK (Yu et al., 2015). Neopeucedalactone, a pyranocoumarin isolated from *P. praeruptorum* roots, was found to inhibit the growth of human leukemic HL-60, prostate cancer PC-3, and THP-1 cell lines *in vitro* (Li et al., 2020).

### 6.4 Neuroprotective activity

One study demonstrated that 24 h of treatment with praeruptorin C (0, 1, and 10 μmol/L) could partially reverse the upregulated expression of GluN2B-containing N-methyl-D-aspartate receptors, inhibit neuronal apoptosis, and balance Bax and Bcl-2 expression. Although it was suggested that praeruptorin C exerted its neuroprotective effects by reversing intracellular Ca^2+^ overload, it is possible other pathways or mechanisms were involved (Yang et al., 2013). In another study, praeruptorin C (1.5, 3.0 mg/kg) was found to alleviate depressive behavior, motor deficit, and neuronal excitotoxicity in 3-nitropropionic acid (3-NP)-treated mice via upregulating the expression of HTT, DARPP32, and BDNF in striatum tissue. Motor behavior was tested using the open field test and rotarod test, while psychiatric symptoms were tested using the forced swimming test and tail suspension test. We suggest that, based on these findings, praeruptorin C may prove therapeutic for cognitive, psychiatric, and movement disorders associated with Huntington’s disease (Wang et al., 2017).

### 6.5 Anti-osteoclastogenic activity

Osteoporosis results in an elevated risk of fracture, compromised bone strength due to low bone density, and metabolic defects. Bone homeostasis depends on the resorption of bone by osteoclasts and formation of bone by osteoblasts. Imbalance of this tightly coupled process can cause diseases such as osteoporosis (Chen et al., 2018). In one study, exposure of RAW264.7 cells for 4 h to praeruptorin C (0, 20 μmol/L) reduced osteoclast formation by obstructing the JNK and NF-κB pathways, without disturbing the p38 and ERK pathways. In ovariectomized (OVX) mice, a model for post-menopausal bone loss, praeruptorin C was found to increase bone mass and decrease osteoclast activity. The antiresorptive properties of praeruptorin C suggest that it may be an effective treatment for osteoporosis, although further research is required (Liu et al., 2017). In bone marrow-derived macrophages, praeruptorin A (10 μmol/L) treatment for 30 min inhibited RANKL-stimulated osteoclast differentiation and p38 and Akt signaling (Yeon, Jeong-Tae, et al., 2014). Praeruptorin D could promote the osteogenic differentiation and proliferation of inflammatory periodontal membrane cells, and upregulate osteogenic gene (*RUNX-2*, *ALP*, and *OCN*) expression at doses of 10, 20, 30, and 40 μg/mL by using RT-qPCR and Alizarin red S staining (Yu, 2022).

### 6.6 Antidepressant activity

Clinical depression is characterized by sustained depressive mood and cognitive dysfunction, including restlessness, anhedonia, sleep disorders, guilt, and repeated thoughts of suicide (Liu et al., 2011). The occurrence of depression is closely related to damage to hippocampal neurons. Chronic stress can damage the hippocampus, resulting in atrophy, apoptosis, or reduced regeneration of hippocampal neurons (Wang and Xu, 2014). Dl-praeruptorin A can protect the nervous and cardio-cerebrovascular systems. For example, 28 days of treatment with Dl-praeruptorin A (10, 30, 60 mg/kg) improved the synaptic ultrastructure of hippocampal CA_1_ region and increased neurotrophic factors and nerve growth factors in the hippocampus (Wang and Xu, 2014). In addition, 28 days of treatment with IMP (15, 30 mg/kg) significantly enhanced 5-HT_1A_R expression, 5-HT level, and sucrose preference; increased the incidence of grooming, rearing, and crossing behaviors; reduced immobility; and decreased 5-HTT expression. These results indicate that IMP exhibits antidepressant effects in rats, likely due to changes in the concentration of 5-HT and 5-HTT, and in the expression of 5-HT_1A_R, in the hippocampus and prefrontal cortex (Zheng et al., 2019). However, the mechanism responsible for the antidepressant activity of *P. praeruptorum* extracts remains unknown, and clinical pharmacological experiments are lacking.

### 6.7 Other activities

*P. praeruptorum* has been shown to exhibit other therapeutic activities, including ventricular remodeling, inhibiting hepatic microsomal drug-metabolizing enzymes, ameliorating memory disruption, and alleviating ischemia/reperfusion injury. Administration of *P. praeruptorum* alcohol extracts to rats for 28 days affected ventricular remodeling and apoptosis-related proteins in different ways, and had a positive influence on ventricular remodeling (Tu et al., 2004). Treatment of mice with total coumarins (50, 100, 200 mg/kg) prolonged the hypnotic duration of pentobarbital sodium in a dose-dependent manner, inhibited the activities of aniline hydroxylase and aminopyrine N-demethylase, minimally influenced the hypnotic effect of barbital sodium, and inhibited the activity of hepatic microsomal drug-metabolizing enzymes (Wang et al., 2004). In addition, administration of nodakenin (10 mg/kg) reduced scopolamine-induced cognitive impairments associated with the Y-maze test and passive avoidance test, as well as minimized escape latency in the Morris water maze test. Nodakenin has been shown to block acetylcholinesterase activity in a dose-dependent manner *in vitro* (Kim et al., 2007). Administration of Dl-praeruptorin A (0.5, 1.0, 2.0 mg/kg) reduced the levels of bcl-2, bax, Fas, and IL-6, and raised the bcl-2/bax ratio, under hypotension without bradycardia. A positive, linear correlation has been demonstrated between bax, Fas, bcl-2, and IL-6, where neutrophil infiltration was minimal. Dl-praeruptorin A was also found to prevent postischemic cell death in rat heart, likely due to the automodulation of immediate-early gene expression of bax, Fas, bcl-2, and IL-6 during myocardial ischemia/reperfusion (Chang et al., 2003). In rats, praeruptorin C (5, 15, 30 mg/kg) was found to reduce ischemia/reperfusion injury induced by coronary ligation, most likely by reducing oxygen free radicals (Liu and Wang, 2009).

## 7 Pharmacokinetic studies

Praeruptorins A, B, and C, the primary metabolites of *P. praeruptorum*, exhibit diverse biological activities, including neuroprotective, antitumor, anti-inflammatory, immunoregulatory, anti-osteoclastogenic, and antidepressant effects. Recently, researchers developed a liquid chromatography–selected ion monitoring–mass spectrometry (LC–SIM–MS) method to conduct a pharmacokinetic study of WaiGan KeSou Formula decoction administered to rats at a dose of 20 mL. The monarch drug of WaiGan KeSou Formula was Qianhu, and its index compound was Praeruptorin A. Within 24 h, the peak concentration (C_max_) of praeruptorin A in plasma was 172.697 ± 17.254 ng/mL, the peak time was 1.50 h, the elimination half-life (t_1/2_) was 1.02 h, the mean retention time was 3.42 h, the AUC_0–τ_ (area under the curve) was 504.866 ± 50.317 h ng/mL, and the AUC_0–∞_ was 514.401 ± 36.950 h ng/mL (He and Wu, 2021). In addition, LC-MS/MS was utilized to evaluate the plasma concentrations of praeruptorin A in rats after a single intragastric dose of 8 g/kg body weight. The researchers found that praeruptorin A was detectable up to 24 h after administration, with an AUC_0–t_ of 311.80 ± 42.38 ng h/mL, a C_max_ of 31.09 ± 4.84 ng/mL, and a t_1/2_ of 7.52 ± 1.00 h (Zhou et al., 2015).

A sensitive, selective, and rapid online solid phase extraction-chiral LC–MS/MS method was developed to conduct a pharmacokinetic study of praeruptorins B and C after orally administering *P. praeruptorum* extract to rats. Praeruptorins B and C were detectable in rat plasma up to 24 h after administration, with AUC_0–t_ values of 187.29 ± 15.02 (B) and 91.64 ± 9.37 h ng/mL (C), C_max_ values of 19.66 ± 4.25 (B) and 7.59 ± 1.98 ng/mL (C), and t_1/2_ values of 8.20 ± 1.21 h (B) and 14.97 ± 3.66 h (C) (Zhou et al., 2015). Nonetheless, additional pharmacokinetic studies should be conducted on the other bioactive metabolites present in *P. praeruptorum*, including praeruptorin D, praeruptorin E, qianhucoumarin B, and praeroside I.

## 8 Quality control

Qianhu is typically processed by washing and immediately drying at low temperature, which must be kept below 60°C. Fresh slices should be thicker than 6 mm (Ren et al., 2021). To maintain medicinal quality, the Chinese Pharmacopoeia dictates the use of microscopic, morphological, HPLC, and TLC detection and identification, as well as ethanol extraction and cold-dipping. By utilizing cold-dipping, the ethanol extract must be more than 20.0% for *P. praeruptorum*. According to the requirements of the Chinese Pharmacopoeia (Chinese Pharmacopoeia Committee of People’s Repulic of China, 2020), the moisture content (after drying) must not exceed 12.0% and the ash content must not exceed 8.0%. Moreover, different extraction methods have different effects on the index metabolites of *P. praeruptorum* (praeruptorins A and B). The reflux method is favored for the extraction of praeruptorin A, producing a much higher praeruptorin A content than ultrasonic methods. Conversely, the ultrasonic method is favored for the extraction of praeruptorin B, producing a much higher praeruptorin B content than the reflux method (Xu et al., 2022). However, it is inadvisable to utilize only one crude, quantitative marker when assessing the quality of *P. praeruptorum* extracts. An array of bioactive metabolites has been detected in *P. praeruptorum* by HPLC, UV, gas chromatography (GC), NMR, high-speed counter-current chromatography coupled with electrospray ionization multi-stage MS (prep-HSCCC/ESI-MS(n)) (Zhou et al., 2015).

The medicinal quality of *P. praeruptorum* is affected by the altitude at which it is produced. According to reports by Luo et al. (2022), an altitude of 900 m improved the praeruptorin A content, while an altitude of 650 m improved the praeruptorin B content. Moreover, the influence of altitude was greater on praeruptorin B content than on praeruptorin A content (Luo et al., 2022). In another study, praeruptorin A and B contents were higher in plants cultivated at high altitudes than in plants cultivated at low altitudes by using HPLC-DAD method to determine the contents of praeruptorin A and B in the 24 batches of Qianhu from different producing areas. According to the experimental results, cluster analysis and principal component analysis were carried out (Yang et al., 2021). In addition, the key climatic factors affecting the praeruptorin content are average relative humidity, average maximum temperature in July, average annual temperature, and average temperature in July (Xu et al., 2021). However, the relationships between Qianhu quality and climatic factors have not been widely investigated, and warrant further study.

Praeruptorin A and B contents are also influenced by plant organ, harvesting time, cultivation environment, and fertilization strategy. According to reports by Li et al. (2022), the HPLC method was used to determine the content of praeruptorin A and B in the cultured *P. praeruptorum* at different harvesting periods, as well as to analyze the fluctuation of praeruptorin A and B at different harvesting periods. Praeruptorin A and B contents are highest in *P. praeruptorum* roots, followed by stems, and are lowest in leaves. In the 14 samples, the praeruptorin A content was found to be much higher in 2-year-old *P. praeruptorum* than in 1-year-old *P. praeruptorum*. Moreover, plants cultivated on southern slopes exhibited an approximately 80% higher content of praeruptorin A than plants grown on slopes of other orientations. However, no significant differences in the contents of praeruptorin A or B were observed in *P. praeruptorum* harvested before or after bolting (Li et al., 2022). Finally, the reasonable application of P and K fertilizers has been found to improve both the yield and medicinal quality *P. praeruptorum*, although the application of N fertilizer should be controlled (Zhou et al., 2022).

## 9 Safety

Praeruptorin C exerted no toxicity on primary cultures of mouse neurons at doses of 0 and 10 μg/mol (Yang et al., 2013). Moreover, an emulsion of praeruptorin C did not exert any toxicity or induce any behavioral changes at doses of 5 and 40 μg/mol in OVX mice by performing a CCK-8 assay (Liu et al., 2017). In BMMs, praeruptorin A was found to be atoxic at doses under 10 mmol/L, but significantly cytotoxic at doses over 20 mmol/L (Yeon et al., 2014). In HepG2 cells, praeruptorins A and C were found to be atoxic at doses of 10 and 100 μg/mol, respectively (Zhou et al., 2013). However, a maximum dose of 200 μg/mol dramatically increased cell toxicity. The toxicity of praeruptorin B on SK-OV-3 ovarian cancer cells was not obvious at doses of 20 and 60 μmol/L (Xue et al., 2021). HCC cell line and normal liver THLE-2 cells were treated with different concentrations of praeruptorin A (0, 10, 20, 30, 40 μg/mL) for 24 h. The cell viability was assessed through 3-(4,5-Dimethylthiazol-2-yl)-2,5-diphenyl-tetrazolium bromide assay. The results implied that praeruptorin A did not induce cytotoxicity in an HCC cell line at doses of 10 and 40 μg/mL (Yu et al., 2021). In addition, praeruptorin A was atoxic to normal liver THLE-2 cells under at concentrations of 10–30 μg/mol. The Chinese Pharmacopoeia 2020 (Chinese Pharmacopoeia Committee of People’s Repulic of China, 2020) recommends a Qianhu dosage of 3–10 g/d. In addition, Qianhu can safely be used with *Pinelliae* Rhizoma, but not with *Gleditsia sinensis* Lam. or *Veratrum nigrum* L. (“Bencaojing Jizhu” 《本草经集注》). Finally, Qianhu is not considered suitable for people with Yin deficiency syndrome (a series of symptoms caused by the deficiency of yin essence or fluid in the human body. Yin: the dark, not active, female principle of the Universe in Chinese philosophy) (Wen, 2023), cough, cold, or cold fluid syndrome. According to cell studies, *P. praeruptorum* appears to be atoxic at a low dose but cytotoxic at higher doses (Zhou et al., 2013; Yeon et al., 2014).

## 10 Conclusion and future perspectives

*P. praeruptorum* is a classical medicinal plant commonly used in TCM preparations. Here, we systematically evaluated the toxicology, molecular mechanisms, pharmacology, phytochemistry, botany, quality control, and traditional uses of *P. praeruptorum* in order to validate the medicinal use of this species. According to the Chinese Pharmacopoeia and classical Chinese botanical drugs, *P. praeruptorum* has been historically prescribed to treat a wide spectrum of diseases including cough, asthma, and pulmonary hypertension. Pharmacological studies suggest that *P. praeruptorum* exhibits anti-inflammatory, expectorant, antitussive, antitumor, neuroprotective, anti-osteoclastogenic, and antidepressant effects, and largely support the traditional uses of this plant. To date, more than 119 distinct phytochemicals have been identified in *P. praeruptorum* extracts, the most common of which are pyranocoumarins and furanocoumarins.

Although there has been considerable progress in evaluating the phytochemistry and pharmacology of *P. praeruptorum*, there are still gaps in our knowledge. First, according to the Chinese Pharmacopoeia 2020 (Chinese Pharmacopoeia Committee of People’s Repulic of China, 2020), Qianhu can disperse wind-heat, reduce cough and phlegm, and dissipate adverse Qi, indicating that Qianhu may alleviate the effects of wind-heat on the lungs (Song et al., 2015). However, most of the active metabolites of *P. praeruptorum* effective against cough and wind-heat are currently administered as crude extracts. Therefore, comprehensive investigations should be conducted to identify the effective metabolites and elucidate their modes of action in order to facilitate clinical trials. Second, we found few reports on the toxicity of *P. praeruptorum* extracts or potential botanical drugs interactions. The potential adverse effects, contraindications, and toxicities of *P. praeruptorum* extracts and their bioactive metabolites should therefore be studied *in vitro*, *in vivo*, and in clinical trials. Third, the majority of the reviewed research was conducted in cell cultures or animal models. Clinical trials in humans will be required to truly evaluate the efficacy *P. praeruptorum* in addressing depression, osteoporosis, cancer, and inflammation, among other diseases. Forth, most of the *P. praeruptorum* containing health products are mainly derived from its root rich in chemical compounds, while non-medicinal parts are rarely exploited. Therefore, it may be interesting to extend the research to the non-medicinal parts of the inexpensive flowers, leaves, and stems of *P. praeruptorum* to ensure the fully utilization of its edible and medicinal values. Finally, new and updated analytical and quality control methods will be required to identify novel markers of quality for the assessment of TCM preparations.

In conclusion, *P. praeruptorum* is rich in medicinal materials, and its pharmacological effects are extensive. With the advantages of modern instruments and data analysis technology in identifying chemical components and separation, Qianhu medicinal materials can be better developed and new drug discovery (Liu, 2020). Future research should be conducted to investigate the mode of action responsible for the pharmacological activities of *P. praeruptorum* extracts, as well as to comprehensively evaluate the potential toxicities, adverse effects, and contraindications of this botanical drugs. Alongside updated quality control measures, these investigations will facilitate clinical trials. Updated *in vivo* pharmacological studies must be performed to validate the traditional uses of *P. praeruptorum*. Finally, the clinical safety and efficacy of *P. praeruptorum*-derived phytochemicals in the treatment of depression, osteoporosis, cancer, and other diseases, require validation.

## Abbreviations

BALF, bronchoalveolar lavage fluid; LPS, lipopolysaccharide; CFA, complete Freund’s adjuvant; ER, erlotinib-resistant; OVX, ovariectomized; HCC, hepatocellular carcinoma; HR-ESI-MS, high-resolution-electrospray ionization-mass spectrometry; Ig, immunoglobulin; IL, interleukin; IMP, imperatorin; IR, infrared spectroscopy; LC–MS/MS, liquid chromatography-mass spectrometry/-mass spectrometry; LC–SIM–MS, liquid chromatography-selected ion monitoring-mass spectrometry; MS, mass spectrometry; NMR, nuclear magnetic resonance; NO, nitric oxide; NSCLC, epidermal growth factor receptor; NSCLC, non-small-cell lung cancer; RCC, renal cell carcinoma; RT-qPCR, real-time fluorescence quantitative polymerase chain reaction; SIMS, secondary ion mass spectroscopy; TCM, traditional Chinese medicine; TNF-α, tumor necrosis factor αtumor necrosis factor α; UV, ultraviolet.
